# Suppression of microRNA168 enhances salt tolerance in rice (*Oryza sativa L.*)

**DOI:** 10.1186/s12870-022-03959-1

**Published:** 2022-12-03

**Authors:** Jiong Wan, Shujun Meng, Qiyue Wang, Jiawen Zhao, Xiaoqian Qiu, Liangfa Wang, Juan Li, Yuan Lin, Liqin Mu, Kuntai Dang, Qiankun Xie, Jihua Tang, Dong Ding, Zhanhui Zhang

**Affiliations:** 1 National Key Laboratory of Wheat and Maize Crop Science, College of Agronomy, Henan Agricultural University, 450002 Zhengzhou, China; 2 Hebi Academy of Agricultural Sciences, 458030 Hebi, China; 3 Institute of Crop Germplasm Resources, Guizhou Academy of Agricultural Sciences, 550006 Guiyang, China; 4 The Shennong laboratory, 450002 Zhengzhou, China

**Keywords:** Rice, miR168, Salt stress, STTM, Gene regulation cascades

## Abstract

**Background:**

Rice is a salt-sensitive crop. Complex gene regulatory cascades are likely involved in salinity stress in rice roots. microRNA168 (miR168) is a conserved miRNA among different plant species. It in-directly regulates the expression of all miRNAs by targeting gene *ARGONAUTE1(AGO1)*. Short Tandem Target Mimic (STTM) technology is an ideal approach to study miRNA functions by in-activating mature miRNA in plants.

**Results:**

In this study, rice miR168 was inactivated by STTM. The T3 generation seedlings of STTM168 exhibited significantly enhanced salt resistance. Direct target genes of rice miR168 were obtained by in silico prediction and further confirmed by degradome-sequencing. *PINHEAD (OsAGO1)*, which was previously suggested to be a plant abiotic stress response regulator. RNA-Seq was performed in root samples of 150mM salt-treated STTM168 and control seedlings. Among these screened 481 differentially expressed genes within STTM168 and the control, 44 abiotic stress response related genes showed significant difference, including four known salt-responsive genes.

**Conclusion:**

Based on sequencing and qRT-PCR, a “miR168-*AGO1*-downstream” gene regulation model was proposed to be responsible for rice salt stress response. The present study proved miR168-AGO1 cascade to play important role in rice salinity stress responding, as well as to be applied in agronomic improvement in further.

**Supplementary Information:**

The online version contains supplementary material available at 10.1186/s12870-022-03959-1.

## Background

During the development of crops, including rice, which need to cope with various environmental stresses. For instance, high salinity is a main limiting factor of plants growth and thereby results in crop yield reduction. As exposed to a high salinity environment, photosynthesis, protein synthesis, and energy metabolism are affected, which can cause crops physiological drought, ion toxicity, and physiological and metabolic disorders, eventually causing cell apoptosis and plant death [1]. During evolution, plants have generated multiple responding mechanisms to salinity stress. The plant prefers to avoid or minimize the effect of salinity stress for keeping the cell alive [2]. Genome-wide detection of high salinity stress-responsive genes has been reported in divergent plant species [3, 4] to reveal that plant salt stress responses and tolerance occur at both the transcriptional and post-transcriptional levels [5, 6]. The increased studies have identified transcription factors and regulatory RNAs to play important roles in abiotic stress response, including salinity stress.

MicroRNAs (miRNAs) were defined as endo-genome small non-coding RNAs that are 21–24 nt in length [7]. It is now clear that miRNA-mediated gene regulation in plants plays an important role in the response to abiotic stress [8, 9], such as drought, salt, high- and low-temperature, and heavy metal stress. Reports on the response of plant miRNAs to drought stress and salt stress, such as miR159, miR171, and miR394 respond to drought stress in Arabidopsis [10]; miR170, miR172, and miR408 are identified to be related to drought stress in rice [11]; Furthermore, miR161 is associated with salt stress in Arabidopsis [12]; miR2111 is involved in the regulation of salt tolerance in soybean [13]; miR156, miR160, miR164, miR167, miR528, and their target genes are also involved in the salt stress response in rice [14, 15]. 11 down-regulated and 3 up-regulated miRNAs were identified in Solanum plants under salt stress [16].

In rice, a recent study proved that suppression of rice miR168 could improve yield, flowering time, and immunity via the regulation of miR535, miR1320, and miR164 by AGO1 [17]. And, miR168 was also identified to express differentially under salt stress conditions by genome-wide miRNA-seq analysis [18]. In maize and wheat, differential miR168 expression was also detected in high salinity conditions [19, 20]. These results indicated that miR168 expression may be associated with plant salt stress responses. However, the roles of miR168, together with the cascade including its direct target genes in salt stress response, were not yet fully uncovered. The increasing studies indicate STTM is an efficient artificial miRNA target mimic technique that allows ones to explore the functions of individual miRNAs [21].

In this study, STTM168 was constructed and transformed into rice. As subjected to salinity stress treatment, STTM168 mutants displayed increased plant heights and root lengths compared with that in the control, indicating that STTM168 seedlings have an enhanced salt tolerance [2]. To explain the miRNA 168-dependent salt tolerance mechanism and regulation pathway, transcriptome sequencing, degradome-seq, and qRT-PCR were performed. The homeostasis within miR168 and its target gene OsAGO1a may regulate rice salt stress responses through their effect on the cellular balance of Na^+^ and further affect plant growth.

## Results

### Functional blockage of rice miR168 mediates enhanced salinity tolerance

**Fig. 1:**
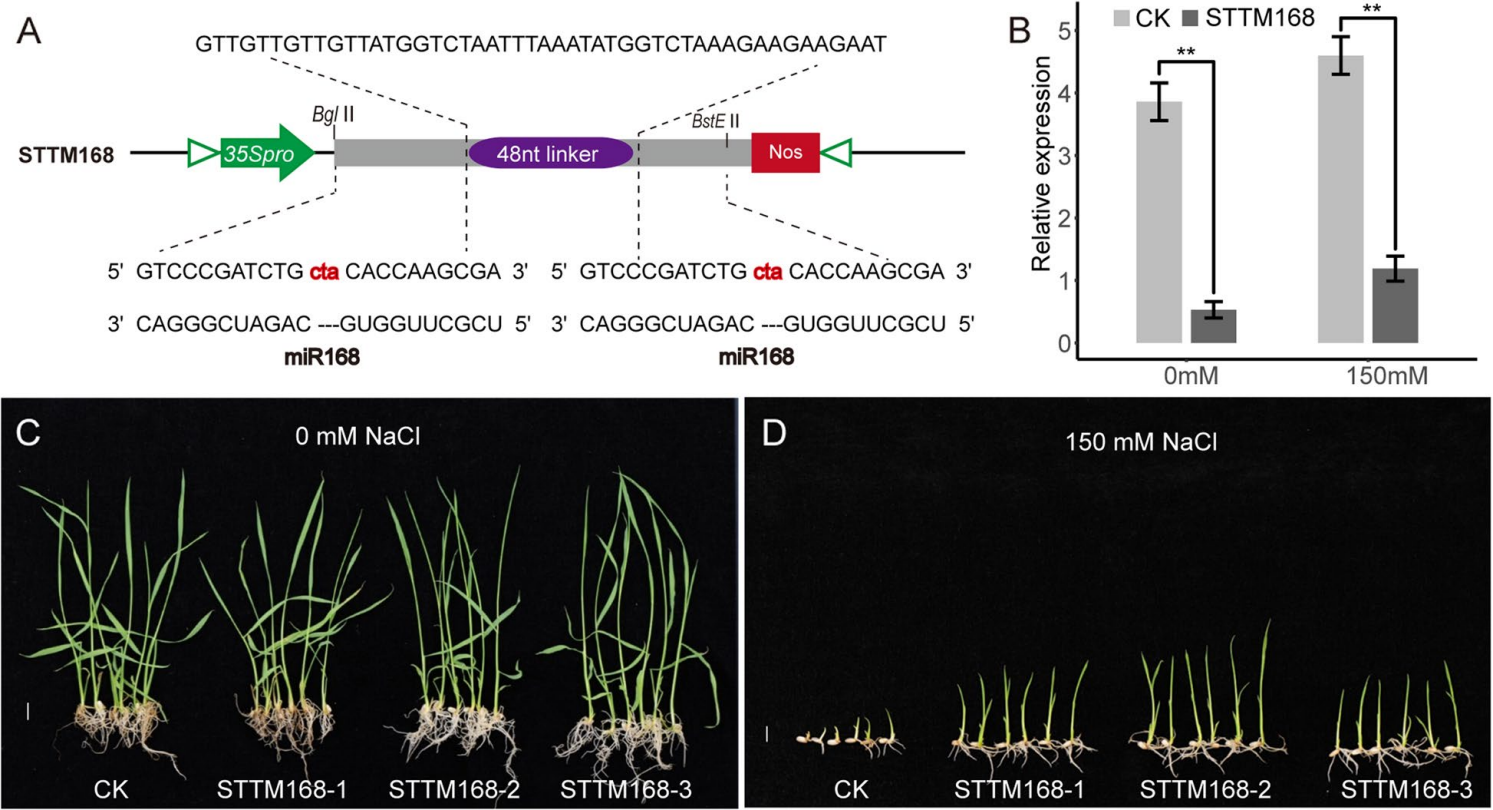
miR168 suppression mediates enhanced salinity stress tolerance in rice. **A** The diagram of STTM168 structure with a 48 nt length spacer flanked by two non-cleavable; (**B**) miR168 expressed alterations mediated by salinity stress in STTM168 transgenic lines. **C**-**D** Performance of the STTM168 and the control seedlings grown on 1/2 MS medium supplemented with or without 150 mM NaCl solution. The scale bar indicates 1 cm

**Fig. 2:**
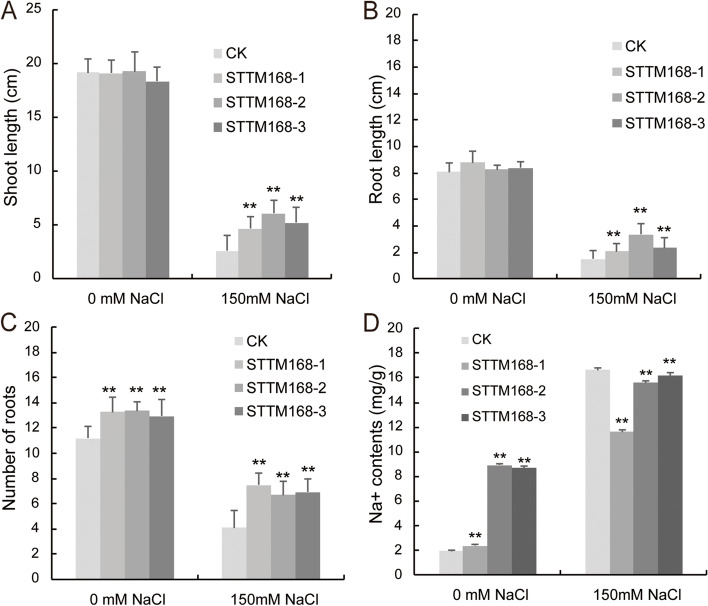
Phenotypic alterations of STTM168 plant and the control under salinity stress. **A **The shoot length of STTM168 and CK seedlings grown on 1/2 MS medium supplemented with or without 150 mM NaCl at 21 d after planting; (**B**) The root length of STTM168 and CK seedlings grown on 1/2 MS medium supplemented with or without 150 mM NaCl at 21 d after planting; (**C**) The root number of STTM168 and CK seedlings grown on 1/2 MS medium supplemented with or without 150 mM NaCl at 21 d after planting; (**D**) Na^+^ content in the roots of STTM168 and CK seedlings grown on 1/2 MS medium supplemented with or without 150 mM NaCl at 21 d after planting. **A**-**D** The values presented in the charts are the mean ± SD (*n* = 3). * t-test *P* value ≤ 0.05; ** t-test *P* value ≤ 0.01

Based on the previous study [21], STTM168 was designed and constructed in pCAMBIA3301 plasmid (Fig. 1 A). By genetic transformation in rice, STTM168 mutant plants were obtained. After genotyping and reproduction, three independent lines were chosen randomly, named STTM168-1, STTM168-2, and STTM168-3, and used in further phenotypic analyses. Compared with the control, the miR168 expression level decreased significantly in STTM168 plants (Fig. 1B). Without salinity stress, there was no significant difference in shoot height and root length between STTM168 plants and the control (Fig. 1 C; Fig. 2). Compared with the CK plants, there was a significant increase in shoot length, root length, and root number in STTM168 plants grown on 1/2 MS medium supplemented with 150 mM NaCl (Figs. 1D and 2). Thereby, STTM168 plants showed enhanced tolerance to salinity compared to the control.

### Identification of the target genes of miR168

**Fig. 3:**
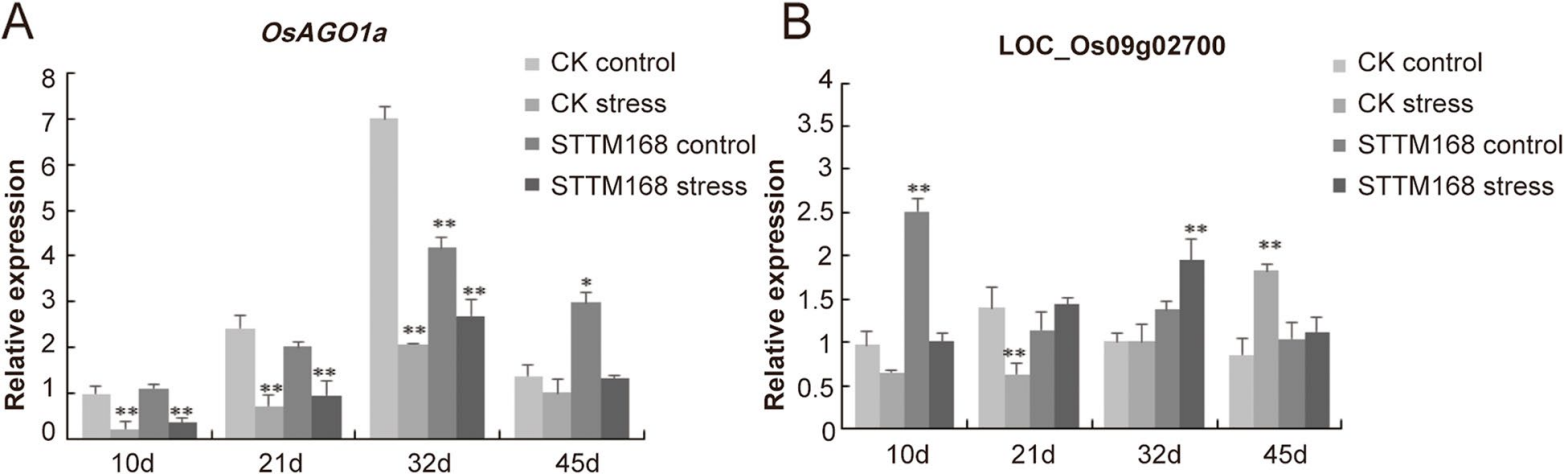
The Expression profiles of miR168 target genes. **A**, **B** The relative expression of LOC_Os02g45070 and LOC_Os09g02700 in STTM168 and the control seedlings grown on 1/2 MS medium under regular condition and salinity stress. Values were normalized to the *OsActin* level. The values presented in the charts are the mean ± SD (*n* = 3). * t-test *P* value ≤ 0.05; ** t-test *P* value ≤ 0.01

The relative expression of miR168 was downregulated in the STTM168 lines compared with CK (Fig. 1B). To further confirm the binding of miR168 to its target genes, degradome sequencing was performed. Two candidate genes were identified, such as *OsAGO1a* (LOC_Os02g45070) and LOC_Os09g02700. During salinity stress treatment, *OsAGO1a* showed down-regulated expression in STTM168 lines. From salinity stress treated for 10 days to 32 days, the expression of *OsAGO1a* exhibited an increasing trend but decreased at treated 45 days (Fig. 3 A). On the other hand, LOC_Os09g02700 exhibited a different expression trend during salinity stress treatment (Fig. 3B). These results indicated that miR168 is a major regulator of the expression of its target gene *OsAGO1a*. As the *AGO1* gene is a key component of an RNA-induced silencing complex (RISC), a significant change in its expression level may lead to large changes in downstream genes, including a vast majority of miRNA families and their respective cascades.

### Salt response and stress response genes identified by RNA-seq and qRT-PCR analyses

**Fig. 4:**
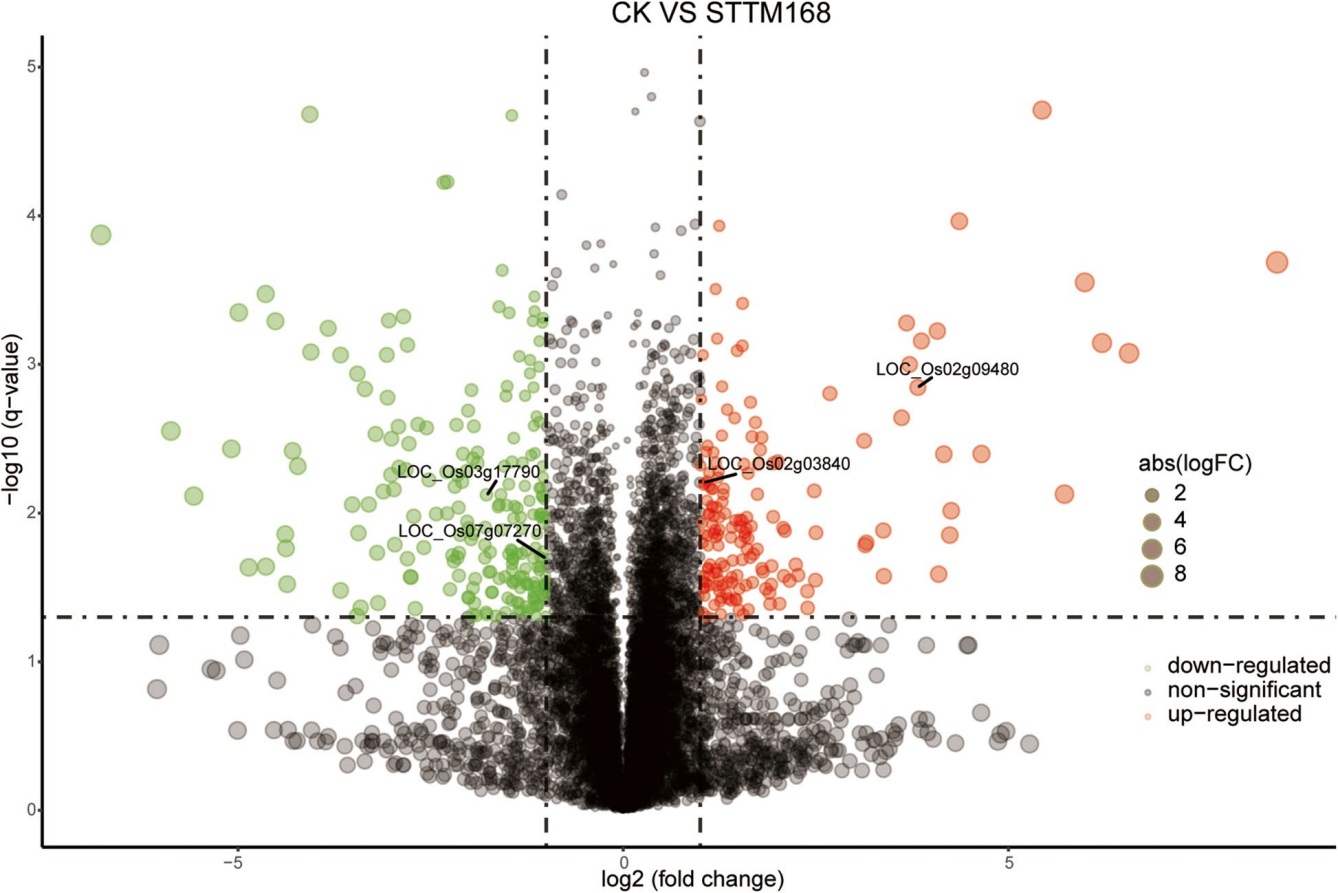
The differentially expressed genes of RNA-seq. Red dots indicate up-regulated genes and green dots for down-regulated genes (Significant level: Padj < 0.05, Log2FoldChange > 1).

To identify the downstream genes regulated by *OsAGO1*, salinity-stressed STTM168 and the control seedlings were subjected to RNA-seq (Fig. 4). In total, there were 481 differentially expressed genes were screened (Padj < 0.05, Log2FoldChange > 1) (Supplementary Table 2). Overall, 44 out of these 481 differentially expressed genes were marked as being stress-response genes (Supplementary Table 3), which accounts for the most abundant group in the differentially expressed genes. Among these stress response genes, there were four salt stress response genes, LOC_Os02g03840.2 (signal transduction factor), *OsRCI 2–5*(LOC_Os03g17790.1), LOC_Os07g07270.1 (Bric-a-Brac, E3 ubiquitin ligase), and LOC_Os02g09480.1 (MYB transcriptional factor). Among these four genes, the expression of the *MYB* gene was only detected in the non-transgenic control, the signal transduction factor was downregulated in the transgenic lines compared with the control, and *OsRCI 2–5* and the Bric-a-Brac E3 ubiquitin ligase were upregulated in the transgenic lines and the control under salt stress conditions.

**Fig. 5:**
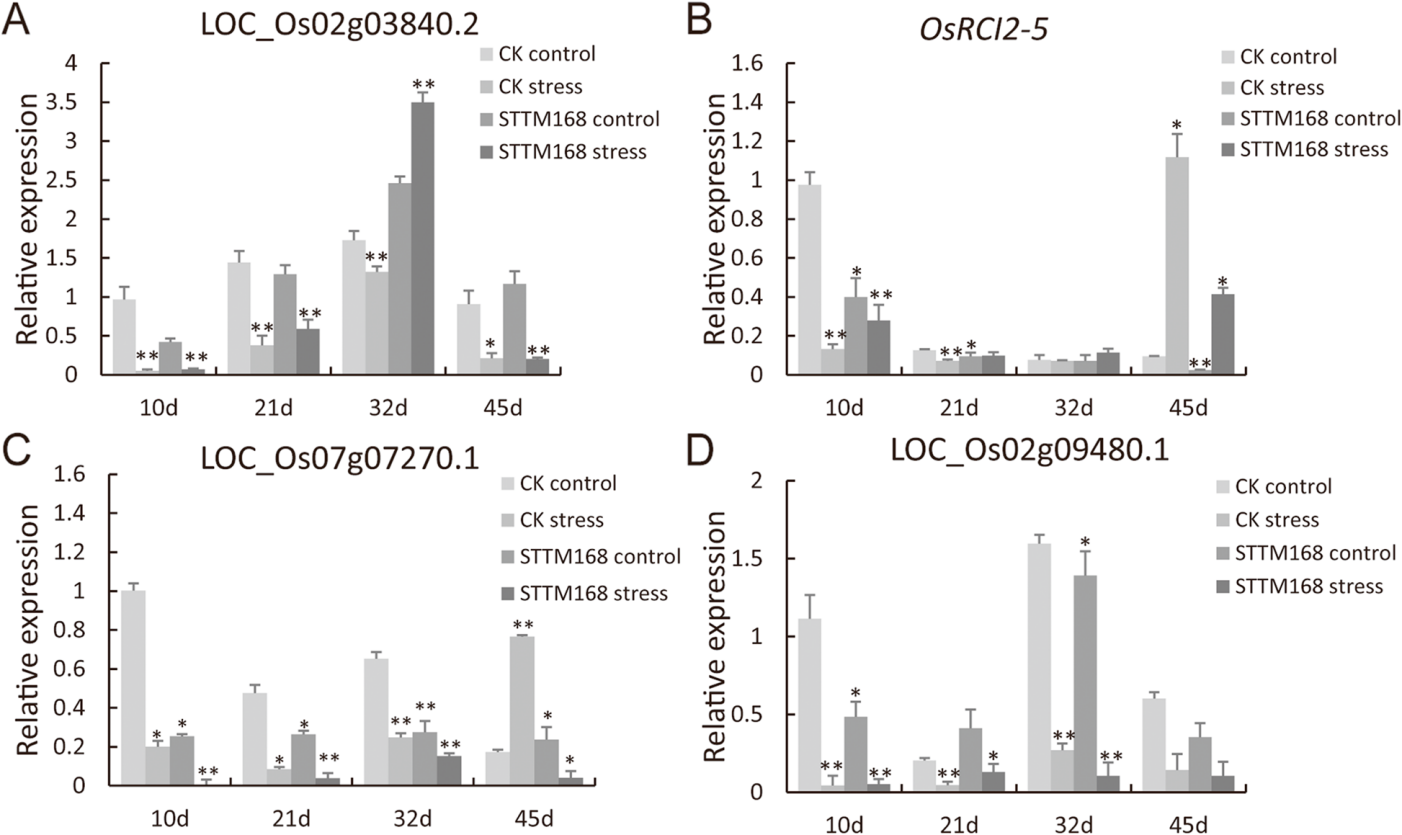
The expression profiles of four selected stress-responsive genes in STTM168 and the control seedlings. **A**-**D** The relative expression of salt-responsive genes in STTM168 and CK seedlings grown on 1/2 MS medium supplemented with or without 150 mM NaCl for 10, 21, 32, or 45 d. E The relative expression of the stress-responsive gene LOC_Os03g50540 in STTM168 and CK seedlings grown on 1/2 MS medium supplemented with or without 150 NaCl for 10, 21, 32, or 45 d. Values were normalized to the *OsActin* level. The values presented in the charts are the mean ± SD (*n* = 3). * t-test *P* value ≤ 0.05; ** t-test *P* value ≤ 0.01

Expression profiles of four stress-responsive genes, including the four annotated as salt-responsive genes, were further validated by qRT-PCR (Fig. 5). The results showed that their expression patterns were consistent with the RNA-Seq data. Based on the above results, we concluded that the above four genes related to salt stress response may be the downstream target genes of *OsAGO1a*.

**Fig. 6:**
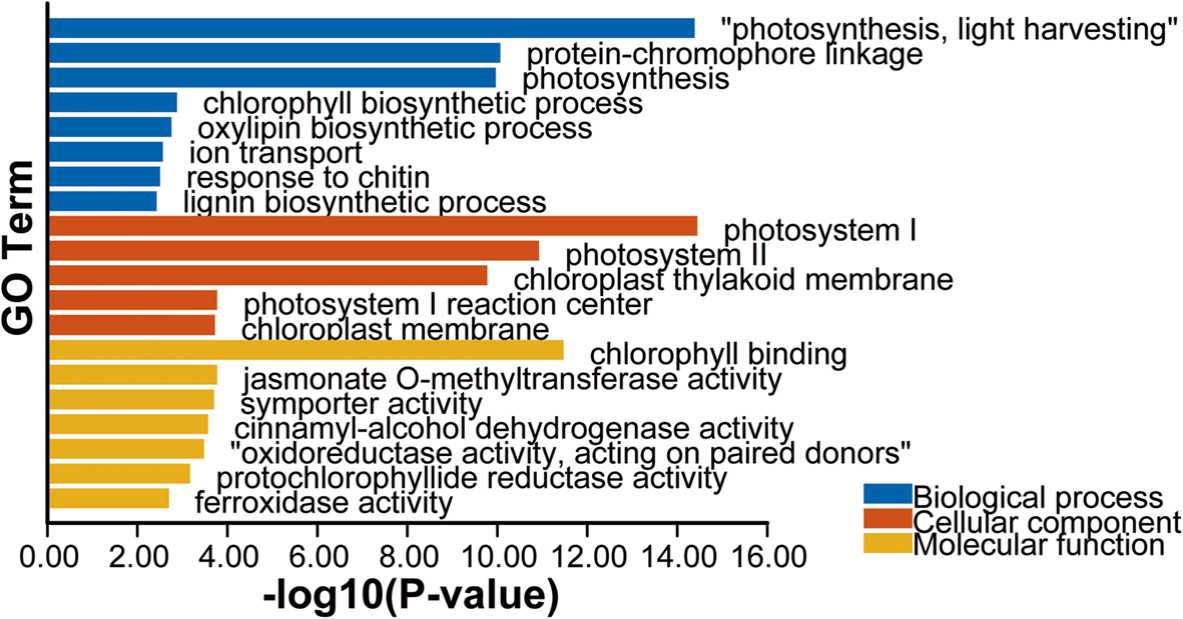
The gene ontology of miR168-dependent differentially expressed genes between the salinity stressed STTM168 and the control. The *p*-values of these genes were less than 0.1. Among them, the chloroplast thylakoid membrane term is the most

**Fig. 7:**
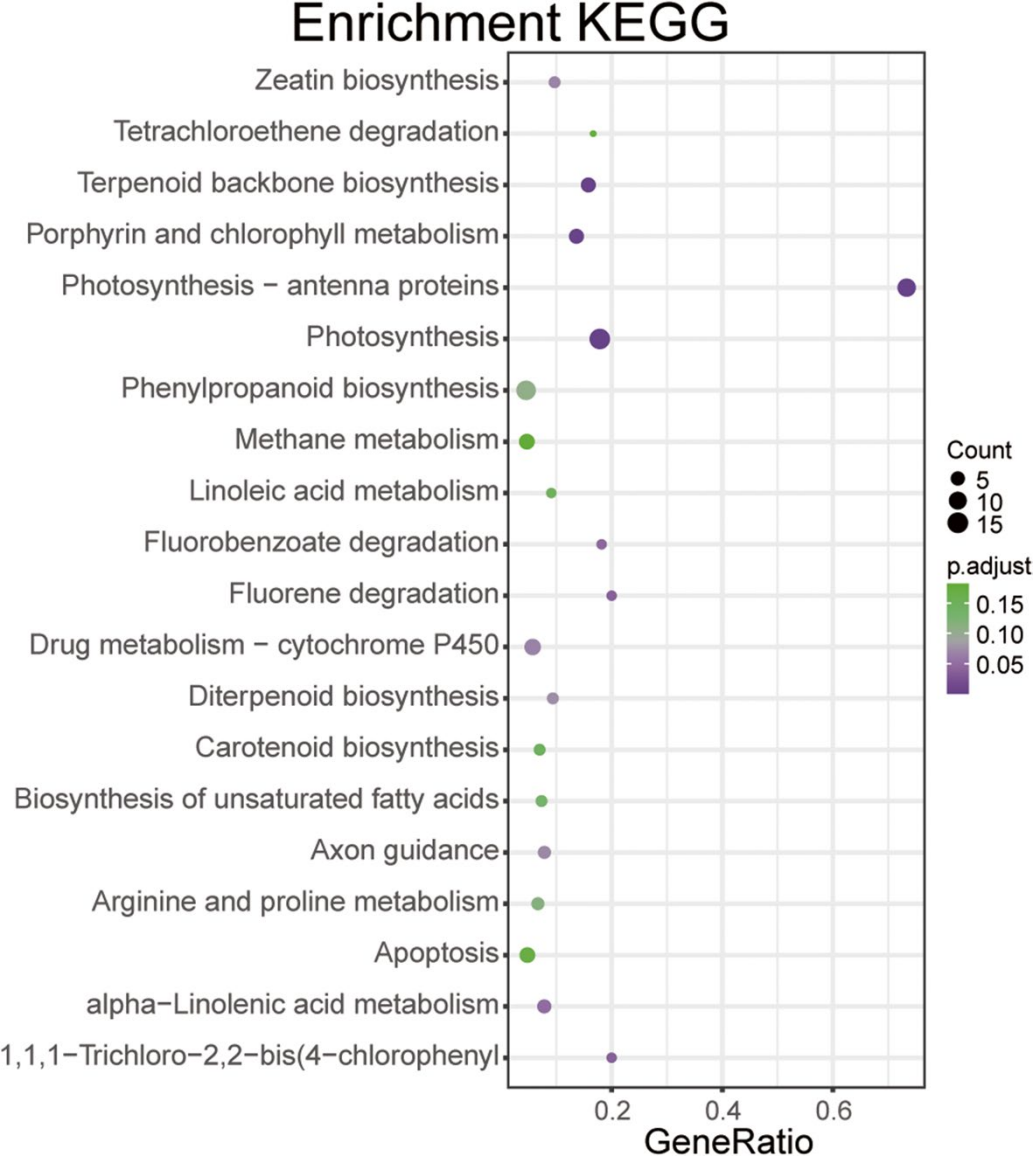
The KEGG analysis of miR168-dependent differentially expressed genes between the salinity stressed STTM168 and the control. The *p*-values of these genes were less than 0.1. Among them, the chloroplast thylakoid membrane term is the most

For defining miR168 regulated gene expression network and pathways, the screened differentially expressed genes between STTM168 and the control were subjected to GO and KEGG enrichment (Figs. 6 and 7). The GO analysis revealed that these genes are strongly associated with photosystem I, chloroplast thylakoid membrane, and transport. In turn, the KEGG enrichment analysis identified these genes to be significantly enriched in terpenoid backbone biosynthesis, porphyrin and chlorophyll metabolism, photosynthesis-antenna proteins, and photosynthesis pathways. Notably, the GO enrichment analysis for the up-regulated differentially expressed genes (DEGs) in STTM168 indicated that the most significant enriched term in the biological process category is related to ion transport, such as transmembrane transport and ion transmembrane transport. And, the up-regulated DEGs were also mainly enriched in integral component of plasma membrane and plant-type cell wall as well as cell periphery (Supplementary Table 4). Similarly, it has been reported that the P-type ATPases localized at the plasma membrane play an essential role in cellular ion transport and further regulate salt tolerance of plants [22]. Collectively, we envision that the up-regulated DEGs in STTM168 involved in ion transport may largely contribute to the salinity tolerance of the STTM168 mutants.

## Discussion

### miR168 regulates salinity stress response via OsAGO1a-associated miRNAs biogenesis

Activation of miRNAs under stress leads to the coherent suppression of many downstream protein-coding genes and physiological responses [23]. The direct target gene of miR168 in rice is *AGO1* (LOC_Os04g47870), which encodes a key protein factor for recruiting miRNAs to the RISC. *AGO1* is also called “Slicer” for its miRNA target gene binding and cutting action. The rice AGO1 was also named *PINHEAD* because the *ago1* null mutant leads to early developmental defects in Arabidopsis [24, 25].

Recently, *AGO1*-dependent transcriptional activation has been reported, possibly via binding to RNA Pol II and/or TATA-box binding proteins mediated by miRNAs [26]. It is possible that miR168, together with its target *AGO1*, may influence downstream gene expression both at the transcriptional and post-transcriptional levels [27–29]. AGO protein has important functions in activating gene expression that differs from classical RNA interference in Arabidopsis. This new function plays an important regulatory role in plant responses to plant hormones and environmental stresses [30].

**Fig. 8:**
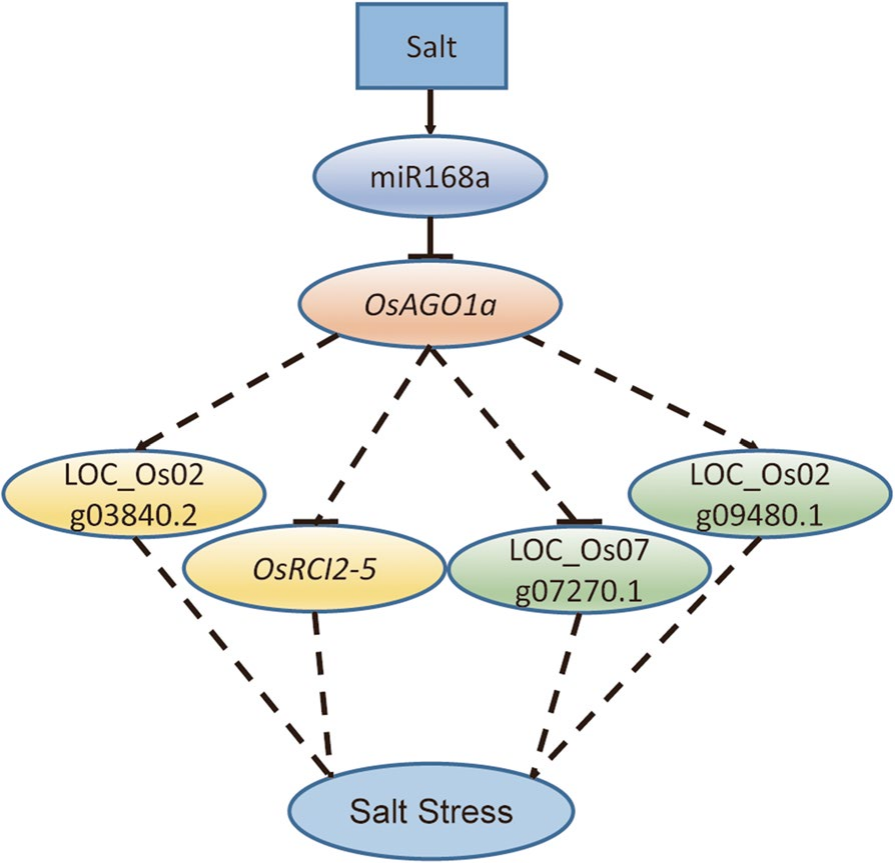
The miR168-directed gene regulation cascade in the response of rice roots to salt stress

Here, we propose the miR168-directed *AGO1*-dependent gene regulation cascade (Fig. 8) to explain the differences in the salt stress response of STTM168 roots and those of the non-transgenic control. It was suggested that miR168 may interrupt salt stress responses by downregulating its target gene *AGO1* to affect the RISC activities of other miRNAs. It has been reported that miR319, which is a positive regulator of salt responses, acts by repressing its target genes at the post-transcriptional level [31]. The main target genes of miRNAs are most commonly transcription factors that may, in turn, bind to downstream gene promoters and control the expression of downstream genes. In this study, the target genes LOC_Os02g03840.2, OsRCI25 (LOC_Os03g17790.1), LOC_Os07g07270.1, LOC_Os02g09480.1, and LOC_Os03g50540 were found to be differentially expressed between the STTM168 and CK plants (Fig. 4). It has been suggested that *AGO1* may influence the Arabidopsis salt stress response through the regulation of miRNAs at the transcriptional and post-transcriptional level [32]. We showed that STTM168 increased rice salt stress tolerance, indicating that the miR168-*AGO1* cascade may be functionally conserved in plant salt stress adaptation. The role of the direct target genes of *AGO1* in the regulation of downstream salt stress responsive genes requires further analysis.

In this study, we also found a gene (LOC_Os09g02700.1) encoding a poly(A)-binding protein. The expression and activity of poly(A)-binding proteins are negatively correlated with the silencing intensity of miRNA, partly because of the antagonistic effect of target mRNA deadenylation [33]. While there is, at present, no evidence of a correlation between polyA and stress responses in plants, they might play a role in salt tolerance, thereby representing a new target gene for miR168.

### Functional genes that may be involved in salt stress response

By analyzing the RNA-seq results, we revealed that there are four genes annotated as “salt stress-responsive” according to their GO terms, including two transcription factors, a gene encoding a membrane protein, and a Bric-a-Brac E3 ubiquitin ligase-coding gene. We speculated that these four genes may be the downstream targets of *OsAGO1a*.

Among the two transcriptional factors, there is a member of the MYB transcription factor family (LOC_Os02g09480.1). MYB proteins are key factors in the abiotic stress response regulatory network [34]. In wheat, 60 *MYB* genes have been identified, 14 of which were induced by salt stress and two that were inhibited by salt stress [35]. It was reported that R2R3-type MYB transcription factors involved in the rice salt stress response function predominately by influencing the signal transduction of ROS [36] and/or transporting ions such as potassium [37]. R2R3-MYB-type proteins play a key role in plant growth, development, and defense response [38].

The other transcription factor-coding gene (LOC_Os07g07270), a homolog of the Arabidopsis *RBB1* gene, serves as a substrate adaptor to the Bric-a-Brac E3 ubiquitin ligase, which belongs to the BTB E3 ligase family. LOC_Os07g07270 was reportedly involved in Arabidopsis salt responses by regulating ERF translation [39]. In the ubiquitination system, the key enzymes mainly include E1 (Ub-activating), E2 (Ub-conjugating), and E3 (Ub-ligase) enzymes. The selective modification of ubiquitin proteins is directed by different families of ubiquitin protein ligases (or E3S). Notably, MYB and BTB cross-talk influence ERF at the transcriptional and post-translational levels [40].

*OsRCI25*, one of the rare cold-inducible genes, was also found to be responding directly to salt stress. This gene was reported to respond to a subset of abiotic stresses including cold, drought, and high salinity. The expression of *OsRCI25* in different stress environments is organ specific [41]. This is consistent with other studies that have shown *OcRCI25* responding to both iron stress and osmotic stress.

### Potential application of STTM168 in rice salinity stress tolerance improvement

Salt stress is important abiotic stress that greatly inhibits crop growth and reduces yields. It is clear that rice is considered to be a salt-sensitive species and salt stress greatly influences the growth, physiological function, and biochemical performance of rice. Therefore, it is considered to be a great challenge to develop superior crop cultivars with both high yields and high salt stress resistance in rice. Discovering favorable genes involved in the balance of rice salt tolerance and identifying key regulators of functional genes could be used to enhance the salt stress resistance in rice. In this study, we found that STTM168 showed significantly enhanced salt resistance compared with the non-transgenic control via OsAGO1a-associated miRNAs biogenesis. These findings may provide excellent rice germplasm with high abiotic stress resistance. This means that the development of superior rice cultivars with high abiotic stress resistance may be possible.

In summary, our research shows that the miR168-*OsAGO1* gene cascade significantly involves in rice salt stress tolerance. Further analysis of RNA-seq data showed that STTM168 transgenic plants significantly improve salt stress tolerance through increasing its target gene expression and other down-stream genes in response. Further researches are required to explore the function of target genes regulated by *OsAGO1*. Considering that the miR168-*AGO* cascade is functionally conserved in plants, it is a promising strategy to improve the abiotic stress tolerance in both rice and other important commercial crops.

## Materials and methods

### STTM vector construction, gene transfer, and transgenic positive plants selection

The STTM vector of rice miR168 was constructed as described previously [42]. Briefly, Briefly, to generate 35 S:STTM168 in the pCAMBIA3301 plasmid, the STTM168 sequence was designed and artificially synthesized. The sequence of STTM168 was then cloned into BglII- and BstEII-digested pCAMBIA3301 plasmid. Further, the constructed plasmid was subjected to transform in Nipponbare (BioRun, Wuhan, China). The obtained T0 generation STTM168 and the non-transgenic control seeds of the same genetic background were planted in the growthroom of Henan Agricultural University (Zhengzhou, China), at an average temperature of 32℃ and 76% humidity in the summer of 2016. Seeds of the T1 generation and the non-transgenic control lines were harvested. The T3 generation of transgenic positive lines and the non-transgenic control seeds were collected for use in the high salinity treatment.

In addition to the BASTA herbicide screening, PCR assays were conducted on the plants at each generation. Genomic DNA was extracted from leaves using the SLS method [43], as described previously. PCR assays were performed with bar gene-specific primers: Bar-F: 5′-ACCCACGTCATGCCAGTTC-3′; and Bar-R: 5′- CTGCACCATCGT CAACCACTA − 3′. Only the herbicide-resistant and PCR-positive seeds were treated as transgenic positives. Finally, we obtained three independent transformation events STTM168-1, STTM168-2, and STTM168-3, respectively.

### Salt treatment and phenotyping

For the salt tolerance assay, 90 hand-selected seeds of the homogeneous transgenic T3 generation and the wild type seeds were surface-sterilized with 70% alcohol for 1 min following sterilization with a mixture of 0.1% HgCl and 2% NaClO (1:1) for 20 min. The seeds were then washed five times with sterilized distilled water before being germinated on 1/2 MS medium (MS salt 2.165 g, sucrose 60.0 g, pH 5.8) supplemented with or without 150 mM NaCl under 28℃/25°C (day/night) during a 12-h photoperiod. Germinated seeds were placed in an incubator with controlled 60% humidity and a 12,000 lx light period (12 h/12 h, light/dark). At 21 d after germination, shoot height, root length, and fresh weight of at least 30 seedlings of each line were measured. The seedlings were then cultured in nutrient solution supplemented with or without 150 mM NaCl and aerated continuously at 32 °C/25°C (day/night) at 12,000 lx light period (14 h/10 h, light/dark), with the nutrient solution changed once a week (pH = 5.5).

The root Na + content was measured as described previously [44]. Briefly, roots were harvested from non-transgenic control and STTM168 seedlings grown on 1/2 MS medium with or without 150 mM NaCl. Samples were washed three times with deionized water, fixed at 105℃ for 30 min, then dried at 70 °C for 3 d. After weighing, the dried samples were digested with 5 ml concentrated sulfuric acid until clarification and the volume increased to 50 ml. The Na^+^ content was measured using an atomic absorption spectrophotometer (ZA3000, HITACHI, Tokyo, Japan).

### Sample collection and RNA extraction

The salt-treated root tissues of 45 days old STTM168 and the non-transgenic control seedlings were harvested for total RNA extraction and RNA-Seq. Total RNA was extracted using Trizol reagent (Invitrogen, CA, USA) following the manufacturer’s instructions. The total RNA quantity and purity were analyzed using a Bioanalyzer 2100 and RNA 6000 Nano LabChip Kit (Agilent, CA, USA) with RIN number > 7.0. Approximately 10 µg of total RNA representing a specific adipose type was subjected to isolate Poly (A) mRNA with oligodT attached magnetic beads (Invitrogen). Following purification, the mRNA was fragmented into small pieces using divalent cations under elevated temperatures. The cleaved RNA fragments were then reverse-transcribed to create the cDNA library following the protocol for the mRNA-seq sample preparation kit (Illumina, San Diego, USA). The average insert size for the paired-end libraries was 300 bp (± 50 bp). Paired-end sequencing was then performed on an Illumina Hiseq2000/2500 (LC Science, Hangzhou, China) following the vendor’s recommended protocol.

### Identification of miR168 target genes in rice

To further focus on candidates, degradome sequencing of developing root samples was performed (LC-Science, Hangzhou, China). First, we constructed a library of samples from the root degradation group. Then, an improved library building process was used for sequencing [45]. According to the plan provided by LC-BIO (Hangzhou, China), single-end sequencing (36 bp) was carried out on an Illumina Hiseq 2500. Differences in the data level of the degradation group were tested by Fisher, Chi-square 2 × 2 test, Chi-square n×n test, standard t-test, and ANOVA analysis of variance. The thresholds were set at 0.01 and 0.05, respectively.

### RNA-seq and data analysis

The adapters and low-quality reads of raw data were filtered using Trimmomatic [46]. The filtered reads were aligned to the rice reference genome (https://plants.ensembl.org/Oryza_sativa/Info/Index) using Hisat2 [47]. The transcripts of each sample were assembled using Featurecounts [48], and the count matrix was generated using a Perl script. The differentially expressed gene analysis was performed using the DESeq2 package in R software [49]. The genes with abs(log2 fold change) > 1 and a statistical significance (p-value < 0.05) were determined to be differentially expressed. The Gene Ontology (GO) annotations were obtained using eggNOG-Mapper v2 (http://eggnog-mapper.embl.de/) [50]. The Kyoto Encyclopedia of Genes and Genomes (KEGG) annotations were obtained on the KEGG website (https://www.kegg.jp/kegg/) [51]. The GO and KEGG enrichment analysis were performed using TBtools [52].

### Relative expression amounts of selected genes were validated by qRT-PCR

The expression profiles of seven selected stress-responsive genes, including *OsAGO1* and *OsAGO2* salt response genes, were obtained by qRT-PCR. RT primers were designed and listed in Supplement Table 1. Relative expression levels of selected miRNAs were measured by stem-loop RT-PCR as described previously [53]. Reverse transcription reactions were then performed with MMLV reverse transcription enzymes (Mir-X miRNA First-Strand Synthesis and SYBR qRT-PCR) to obtain first-strand cDNA. PCR primers (Supplementary Table 1) were designed using Primer3 (http://primer3.ut.ee/) [54]. Each reaction was performed in triplicate and *OsActin* was used as the reference gene. miRNA concentration was set for each reaction with *U6* as the internal control. RT-miR168a, the mRQ 3′ primer supplied with the kit, was also used (Mir-X miRNA First-Strand Synthesis and SYBR qRT-PCR, Clontech).

## Supplementary Information

Additional file 1: **Table S1**. The qRT-PCR primer list.

Additional file 2: **Table S2**. Differentially expressed stress response genes.

Additional file 3: **Table S3**. Stress-responsive genes differentially expressed between the roots of CK and STTM168 plants grown in salt.

Additional file 4: **Table S4**. Distribution of up-regulated DEGs into different categories of Gene Ontology (GO).

## Data Availability

The RNA-seq datasets are available from the National Center for Biotechnology Information, under BioProject number PRJNA873756.

## Abbreviations

STTM: Short Tandem Target Mimic
AGO1: ARGONAUTE1
OsAGO1: PINHEAD
GO: Gene Ontology
KEGG: Kyoto Encyclopedia of Genes and Genomes
qRT-PCR: quantitative Real-time PCR
DEGs: Differentially expressed genes
