# Supplementary material for: Suppression of microRNA168 enhances salt tolerance in rice (Oryza sativa L.)
Source: BMC Plant Biol. 2022 Dec 3;22:563. doi: 10.1186/s12870-022-03959-1 (PMC9719116; doi:10.1186/s12870-022-03959-1)
Supplement: Supplementary file 1 — Table S1. The qRT-PCR primer list. [file 12870_2022_3959_MOESM1_ESM.docx]

**Supplementary Table 1** qRT-PCR primer list.

| Name | Primer sequence (5’-3’) |
| --- | --- |
| RT-miR168a-F | TTGGTGCAGATCGGGACAAAA |
| RT-LOC_Os02g45070-F | AAGGAGGAATGCCACAACAC |
| RT-LOC_Os02g45070-R | GGCCAGTTCTTGATGGTGAT |
| RT-LOC_Os09g02700-F | GCGTGGATTGGGTATTTTGT |
| RT-LOC_Os09g02700-R | AGTTTGCTTCATTTGGTGCAG |
| RT-LOC_Os02g03840.2-F | ACAGGTTCAGGAACGTGGTC |
| RT-LOC_Os02g03840.2-R | TGTGCTGAATTCTGGCTTTG |
| RT-LOC_Os03g17790.1-F | TGCTCCTAGCCATCTTCCTG |
| RT-LOC_Os03g17790.1-R | CCGGGATGTATCCCAGTATG |
| RT-LOC_Os03g50540-F | GATCCCCAAGGGGTTCAC |
| RT-LOC_Os03g50540-R | AGAGACGGCTGTGGTAGCAG |
| RT-LOC_Os07g07270.1-F | TAGCATTGGCTGATCGACAC |
| RT-LOC_Os07g07270.1-R | GGATTGAAGTGATGGGCAGT |
| RT-LOC_Os02g09480.1-F | CTCACCTCGCTGTCCCTCT |
| RT-LOC_Os02g09480.1-R | GCCATGTAGTTGCGGACTTC |
| β-actin-F | GGAAGTACAGTGTCTGGATTGGAG |
| β-actin-R | TCTTGGCTTAGCATTCTTGGGT |
