# Supplementary material for: Suppression of microRNA168 enhances salt tolerance in rice (Oryza sativa L.)
Source: BMC Plant Biol. 2022 Dec 3;22:563. doi: 10.1186/s12870-022-03959-1 (PMC9719116; doi:10.1186/s12870-022-03959-1)
Supplement: Supplementary file 2 — Table S2. Differentially expressed stress response genes. [file 12870_2022_3959_MOESM2_ESM.docx]

**Supplementary Table 2** Differentially expressed stress response genes

| Gene name | Description | log2(fc) | P-val | Q-val |
| --- | --- | --- | --- | --- |
| LOC_Os06g11240 | 12-oxophytodienoate reductase, putative, expressed | 2.39 | 0.04 | 0.48 |
| LOC_Os01g01710 | 1-deoxy-D-xylulose 5-phosphate reductoisomerase, chloroplast precursor, putative, expressed | 1.60 | 0.01 | 0.36 |
| LOC_Os03g45210 | 2-aminoethanethiol dioxygenase, putative, expressed | 1.19 | 0.01 | 0.37 |
| LOC_Os03g50540 | 2Fe-2S iron-sulfur cluster binding domain containing protein, expressed | 1.62 | 0.01 | 0.31 |
| LOC_Os03g61960 | 2Fe-2S iron-sulfur cluster binding domain containing protein, expressed | -2.01 | 0.00 | 0.27 |
| LOC_Os08g01380 | 2Fe-2S iron-sulfur cluster binding domain containing protein, expressed | -1.61 | 0.00 | 0.21 |
| LOC_Os08g40610 | 30S ribosomal protein S16, putative, expressed | -2.43 | 0.01 | 0.36 |
| LOC_Os07g42170 | 60S ribosomal protein, putative, expressed | 5.99 | 0.00 | 0.20 |
| LOC_Os11g47970 | AAA-type ATPase family protein, putative, expressed | -1.27 | 0.02 | 0.42 |
| LOC_Os01g72900 | abscisic stress-ripening, putative, expressed | -1.53 | 0.03 | 0.43 |
| LOC_Os04g54330 | acetyltransferase, GNAT family, putative, expressed | 1.75 | 0.00 | 0.31 |
| LOC_Os10g36650 | actin, putative, expressed | -1.04 | 0.01 | 0.32 |
| LOC_Os10g37670 | actin-depolymerizing factor, putative, expressed | -1.02 | 0.03 | 0.44 |
| LOC_Os01g42830 | adaptin ear-binding coat-associated protein 2, putative, expressed | 1.02 | 0.01 | 0.37 |
| LOC_Os12g01140 | AGC_PVPK_like_kin82y.2 - ACG kinases include homologs to PKA, PKG and PKC, expressed | 1.33 | 0.01 | 0.36 |
| LOC_Os02g52710 | alpha-amylase precursor, putative, expressed | -4.38 | 0.02 | 0.39 |
| LOC_Os03g20420 | alpha-N-arabinofuranosidase A, putative, expressed | 1.65 | 0.02 | 0.41 |
| LOC_Os04g57560 | amine oxidase, flavin-containing, domain containing protein, expressed | 1.74 | 0.01 | 0.34 |
| LOC_Os06g36180 | amino acid transporter, putative, expressed | -1.09 | 0.00 | 0.21 |
| LOC_Os04g56470 | amino acid transporter, putative, expressed | 1.09 | 0.00 | 0.29 |
| LOC_Os12g09300 | amino acid transporter, putative, expressed | 1.46 | 0.01 | 0.36 |
| LOC_Os03g21960 | aminotransferase, putative, expressed | 1.25 | 0.01 | 0.37 |
| LOC_Os08g10510 | aminotransferase, putative, expressed | 2.16 | 0.03 | 0.44 |
| LOC_Os02g56530 | ankyrin repeat domain containing protein, expressed | 2.27 | 0.03 | 0.43 |
| LOC_Os09g27990 | annexin, putative, expressed | 1.66 | 0.03 | 0.44 |
| LOC_Os01g21120 | AP2 domain containing protein, expressed | -1.43 | 0.02 | 0.41 |
| LOC_Os06g10780 | AP2 domain containing protein, expressed | -1.93 | 0.04 | 0.48 |
| LOC_Os04g46400 | AP2 domain containing protein, expressed | -2.50 | 0.02 | 0.40 |
| LOC_Os06g07030 | AP2 domain containing protein, expressed | -4.99 | 0.00 | 0.21 |
| LOC_Os02g51110 | aquaporin protein, putative, expressed | 1.68 | 0.03 | 0.44 |
| LOC_Os06g12310 | aquaporin protein, putative, expressed | 2.09 | 0.03 | 0.43 |
| LOC_Os10g35050 | aquaporin protein, putative, expressed | -1.73 | 0.02 | 0.40 |
| LOC_Os03g60780 | armadillo/beta-catenin-like repeat containing protein, expressed | -1.44 | 0.02 | 0.41 |
| LOC_Os09g28050 | asparate aminotransferase, putative, expressed | 1.87 | 0.04 | 0.48 |
| LOC_Os02g48900 | aspartic proteinase nepenthesin-1 precursor, putative, expressed | 1.12 | 0.03 | 0.43 |
| LOC_Os09g30414 | aspartic proteinase nepenthesin-2 precursor, putative, expressed | -2.70 | 0.04 | 0.48 |
| LOC_Os06g48330 | ATEXO70G1, putative, expressed | 3.68 | 0.00 | 0.21 |
| LOC_Os06g02420 | ATOZI1, putative, expressed | -1.00 | 0.05 | 0.48 |
| LOC_Os11g01010 | autophagy-related protein 8D, putative, expressed | -3.07 | 0.00 | 0.22 |
| LOC_Os04g36054 | auxin response factor 9, putative, expressed | 1.11 | 0.01 | 0.36 |
| LOC_Os11g32110 | auxin response factor, putative, expressed | 1.25 | 0.03 | 0.43 |
| LOC_Os05g41420 | auxin-induced protein 5NG4, putative, expressed | 1.91 | 0.04 | 0.48 |
| LOC_Os08g43270 | BAG domain-containing protein, putative, expressed | 2.39 | 0.03 | 0.45 |
| LOC_Os02g07930 | B-box zinc finger family protein, putative, expressed | 4.26 | 0.01 | 0.36 |
| LOC_Os01g04050 | BBTI12 - Bowman-Birk type bran trypsin inhibitor precursor, expressed | -1.37 | 0.02 | 0.40 |
| LOC_Os08g39380 | beta 1,2-xylosyltransferase, putative, expressed | 2.15 | 0.00 | 0.30 |
| LOC_Os01g38580 | beta,beta-carotene 9,10-dioxygenase, putative, expressed | 1.02 | 0.03 | 0.43 |
| LOC_Os11g31540 | BRASSINOSTEROID INSENSITIVE 1-associated receptor kinase 1 precursor, putative, expressed | -1.31 | 0.01 | 0.37 |
| LOC_Os02g38120 | BTBN3 - Bric-a-Brac, Tramtrack, Broad Complex BTB domain with non-phototropic hypocotyl 3 NPH3 domain, expressed | 1.12 | 0.01 | 0.33 |
| LOC_Os01g14520 | C4-dicarboxylate transporter/malic acid transport protein domain containing protein, expressed | 1.85 | 0.03 | 0.45 |
| LOC_Os02g18880 | calcineurin B, putative, expressed | 1.56 | 0.05 | 0.48 |
| LOC_Os02g18930 | calcineurin B, putative, expressed | 1.30 | 0.01 | 0.34 |
| LOC_Os04g43170 | caleosin related protein, putative, expressed | -2.28 | 0.01 | 0.34 |
| LOC_Os01g72080 | calmodulin-like protein 1, putative, expressed | 6.57 | 0.00 | 0.22 |
| LOC_Os03g19380 | calvin cycle protein CP12, putative, expressed | -3.41 | 0.04 | 0.48 |
| LOC_Os02g03410 | CAMK_CAMK_like.12 - CAMK includes calcium/calmodulin depedent protein kinases, expressed | -1.51 | 0.00 | 0.26 |
| LOC_Os02g41580 | CAMK_CAMK_like.14 - CAMK includes calcium/calmodulin depedent protein kinases, expressed | 1.40 | 0.04 | 0.47 |
| LOC_Os08g42750 | CAMK_CAMK_like.37 - CAMK includes calcium/calmodulin depedent protein kinases, expressed | -3.52 | 0.01 | 0.35 |
| LOC_Os12g08760 | carboxyvinyl-carboxyphosphonate phosphorylmutase, putative, expressed | 1.66 | 0.03 | 0.43 |
| LOC_Os02g42640 | CBS domain-containing protein, putative, expressed | -1.16 | 0.00 | 0.26 |
| LOC_Os06g48590 | CGMC_MAPKCMGC_2_SLT2y_ERK.2 - CGMC includes CDA, MAPK, GSK3, and CLKC kinases, expressed | 1.90 | 0.03 | 0.46 |
| LOC_Os03g18420 | CHCH domain containing protein, expressed | -1.17 | 0.04 | 0.46 |
| LOC_Os03g04060 | CHIT16 - Chitinase family protein precursor, expressed | 1.15 | 0.01 | 0.35 |
| LOC_Os06g51050 | CHIT7 - Chitinase family protein precursor, expressed | -1.72 | 0.02 | 0.42 |
| LOC_Os07g36170 | chitin-inducible gibberellin-responsive protein, putative, expressed | -1.15 | 0.00 | 0.21 |
| LOC_Os01g41710 | chlorophyll A-B binding protein, putative, expressed | -1.44 | 0.02 | 0.41 |
| LOC_Os06g21590 | chlorophyll A-B binding protein, putative, expressed | -1.70 | 0.03 | 0.45 |
| LOC_Os09g17740 | chlorophyll A-B binding protein, putative, expressed | -3.06 | 0.00 | 0.26 |
| LOC_Os08g33820 | chlorophyll A-B binding protein, putative, expressed | -2.08 | 0.00 | 0.30 |
| LOC_Os07g38960 | chlorophyll A-B binding protein, putative, expressed | -1.74 | 0.02 | 0.42 |
| LOC_Os07g37550 | chlorophyll A-B binding protein, putative, expressed | -1.97 | 0.00 | 0.26 |
| LOC_Os07g37240 | chlorophyll A-B binding protein, putative, expressed | -1.91 | 0.05 | 0.48 |
| LOC_Os04g38410 | chlorophyll A-B binding protein, putative, expressed | -2.29 | 0.00 | 0.10 |
| LOC_Os03g39610 | chlorophyll A-B binding protein, putative, expressed | -2.15 | 0.01 | 0.32 |
| LOC_Os11g13890 | chlorophyll A-B binding protein, putative, expressed | -1.78 | 0.05 | 0.48 |
| LOC_Os01g64960 | chlorophyll A-B binding protein, putative, expressed | -1.89 | 0.00 | 0.30 |
| LOC_Os09g25150 | cinnamoyl-CoA reductase, putative, expressed | -1.01 | 0.03 | 0.43 |
| LOC_Os01g35330 | circumsporozoite protein precursor, putative, expressed | -5.09 | 0.00 | 0.30 |
| LOC_Os03g59290 | C-methyltransferase, putative, expressed | -1.66 | 0.01 | 0.36 |
| LOC_Os04g40040 | copper methylamine oxidase precursor, putative, expressed | 1.10 | 0.02 | 0.39 |
| LOC_Os12g34980 | csAtPR5, putative, expressed | 1.48 | 0.00 | 0.22 |
| LOC_Os08g09010 | Cupin domain containing protein, expressed | 1.45 | 0.00 | 0.28 |
| LOC_Os03g57960 | cupin domain containing protein, expressed | -3.83 | 0.00 | 0.21 |
| LOC_Os03g10110 | cupin domain containing protein, expressed | -2.67 | 0.00 | 0.28 |
| LOC_Os03g46100 | cupin domain containing protein, expressed | -3.01 | 0.00 | 0.29 |
| LOC_Os05g25950 | cyclin, putative, expressed | -3.67 | 0.00 | 0.22 |
| LOC_Os07g29760 | cysteine proteinase A494 precursor, putative, expressed | -2.91 | 0.00 | 0.31 |
| LOC_Os01g68660 | cysteine proteinase inhibitor precursor protein, putative, expressed | 3.19 | 0.01 | 0.34 |
| LOC_Os06g30130 | cysteine-rich receptor-like protein kinase 10 precursor, putative, expressed | 1.01 | 0.00 | 0.26 |
| LOC_Os11g44690 | cysteine-rich receptor-like protein kinase 19 precursor, putative, expressed | 1.09 | 0.05 | 0.48 |
| LOC_Os04g56430 | cysteine-rich receptor-like protein kinase, putative, expressed | 1.45 | 0.04 | 0.47 |
| LOC_Os05g02200 | cysteine-rich repeat secretory protein 55 precursor, putative, expressed | -1.35 | 0.00 | 0.22 |
| LOC_Os01g51540 | cytidine/deoxycytidylate deaminase, putative, expressed | 4.10 | 0.03 | 0.43 |
| LOC_Os02g43360 | cytochrome b5-like Heme/Steroid binding domain containing protein, expressed | 1.04 | 0.00 | 0.22 |
| LOC_Os07g37030 | cytochrome b6-f complex iron-sulfur subunit, chloroplast precursor, putative, expressed | -1.09 | 0.00 | 0.28 |
| LOC_Os07g44130 | cytochrome P450 72A1, putative, expressed | 2.03 | 0.04 | 0.48 |
| LOC_Os07g23570 | cytochrome P450 72A1, putative, expressed | -2.12 | 0.01 | 0.38 |
| LOC_Os11g05380 | cytochrome P450, putative, expressed | 2.77 | 0.02 | 0.41 |
| LOC_Os11g18570 | cytochrome P450, putative, expressed | 1.25 | 0.00 | 0.16 |
| LOC_Os01g50490 | cytochrome P450, putative, expressed | 1.88 | 0.03 | 0.45 |
| LOC_Os02g36070 | cytochrome P450, putative, expressed | -3.35 | 0.00 | 0.26 |
| LOC_Os02g38900 | cytosolic Fe-S cluster assembling factor NBP35, putative, expressed | -1.45 | 0.04 | 0.47 |
| LOC_Os06g40020 | DEAD-box ATP-dependent RNA helicase 52A, putative, expressed | -2.01 | 0.01 | 0.37 |
| LOC_Os03g03810 | DEF8 - Defensin and Defensin-like DEFL family, expressed | -3.12 | 0.01 | 0.34 |
| LOC_Os10g21670 | dehydration stress-induced protein, putative, expressed | 1.94 | 0.04 | 0.48 |
| LOC_Os11g26790 | dehydrin, putative, expressed | -1.96 | 0.05 | 0.48 |
| LOC_Os11g40690 | dehydrogenase, putative, expressed | 3.61 | 0.00 | 0.28 |
| LOC_Os08g01760 | dehydrogenase, putative, expressed | 1.06 | 0.04 | 0.46 |
| LOC_Os04g15920 | dehydrogenase, putative, expressed | 1.89 | 0.01 | 0.36 |
| LOC_Os10g29470 | dehydrogenase, putative, expressed | -1.50 | 0.04 | 0.46 |
| LOC_Os08g44210 | dihydroneopterin aldolase, putative, expressed | -1.38 | 0.04 | 0.47 |
| LOC_Os12g12600 | dirigent, putative, expressed | 2.60 | 0.00 | 0.29 |
| LOC_Os03g16350 | DNA binding protein, putative, expressed | 5.44 | 0.00 | 0.06 |
| LOC_Os01g74370 | domain of unknown function DUF966 domain containing protein, expressed | -2.28 | 0.01 | 0.36 |
| LOC_Os05g01730 | drought induced 19 protein, putative, expressed | -1.17 | 0.02 | 0.41 |
| LOC_Os04g15690 | DSBA-like thioredoxin domain containing protein, expressed | 1.18 | 0.01 | 0.38 |
| LOC_Os07g47540 | DUF567 domain containing protein, putative, expressed | 1.69 | 0.02 | 0.40 |
| LOC_Os01g52100 | DUF581 domain containing protein, expressed | -1.07 | 0.01 | 0.33 |
| LOC_Os01g15260 | dynein light chain type 1 domain containing protein, expressed | 1.66 | 0.01 | 0.37 |
| LOC_Os06g04940 | early nodulin 93 ENOD93 protein, putative, expressed | -1.98 | 0.02 | 0.39 |
| LOC_Os12g04690 | embryogenesis transmembrane protein, putative, expressed | 2.89 | 0.03 | 0.46 |
| LOC_Os11g04830 | embryogenesis transmembrane protein, putative, expressed | -2.98 | 0.01 | 0.34 |
| LOC_Os09g36530 | endonuclease/exonuclease/phosphatase family protein, putative, expressed | 1.05 | 0.03 | 0.44 |
| LOC_Os04g09900 | ent-kaurene synthase, chloroplast precursor, putative, expressed | 1.59 | 0.03 | 0.44 |
| LOC_Os04g36760 | enzyme of the cupin superfamily protein, putative, expressed | -1.27 | 0.02 | 0.40 |
| LOC_Os02g43840 | ethylene-responsive element-binding protein, putative, expressed | -1.29 | 0.03 | 0.45 |
| LOC_Os05g31020 | eukaryotic peptide chain release factor subunit 1-1, putative, expressed | -1.11 | 0.04 | 0.48 |
| LOC_Os01g73880 | eukaryotic translation initiation factor, putative, expressed | -1.19 | 0.00 | 0.30 |
| LOC_Os01g10400 | expressed protein | -1.27 | 0.00 | 0.26 |
| LOC_Os10g39980 | expressed protein | 1.16 | 0.01 | 0.35 |
| LOC_Os03g01700 | expressed protein | 1.93 | 0.02 | 0.41 |
| LOC_Os01g09430 | expressed protein | 1.58 | 0.01 | 0.38 |
| LOC_Os04g38790 | expressed protein | -1.33 | 0.05 | 0.48 |
| LOC_Os08g32690 | expressed protein | 1.58 | 0.01 | 0.38 |
| LOC_Os05g02070 | expressed protein | -1.95 | 0.00 | 0.30 |
| LOC_Os04g03164 | expressed protein | 3.38 | 0.01 | 0.37 |
| LOC_Os10g18340 | expressed protein | -1.16 | 0.00 | 0.28 |
| LOC_Os07g01910 | expressed protein | 1.41 | 0.01 | 0.37 |
| LOC_Os07g45490 | expressed protein | 1.24 | 0.02 | 0.39 |
| LOC_Os05g40890 | expressed protein | 1.48 | 0.03 | 0.43 |
| LOC_Os01g73500 | expressed protein | -1.15 | 0.00 | 0.30 |
| LOC_Os08g40940 | expressed protein | 2.24 | 0.02 | 0.41 |
| LOC_Os12g01360 | expressed protein | 1.50 | 0.03 | 0.45 |
| LOC_Os04g35070 | expressed protein | 3.87 | 0.00 | 0.21 |
| LOC_Os06g05480 | expressed protein | -4.64 | 0.00 | 0.21 |
| LOC_Os02g03840 | expressed protein | 1.09 | 0.01 | 0.33 |
| LOC_Os05g48630 | expressed protein | -1.44 | 0.00 | 0.30 |
| LOC_Os06g02370 | expressed protein | 1.05 | 0.01 | 0.33 |
| LOC_Os09g17329 | expressed protein | 1.94 | 0.02 | 0.40 |
| LOC_Os01g45659 | expressed protein | -2.25 | 0.03 | 0.45 |
| LOC_Os05g49670 | expressed protein | 3.07 | 0.00 | 0.21 |
| LOC_Os05g42360 | expressed protein | -1.02 | 0.04 | 0.46 |
| LOC_Os04g32580 | expressed protein | -1.44 | 0.03 | 0.44 |
| LOC_Os08g13710 | expressed protein | 1.00 | 0.05 | 0.48 |
| LOC_Os11g07000 | expressed protein | 1.70 | 0.01 | 0.34 |
| LOC_Os01g49370 | expressed protein | -1.59 | 0.01 | 0.34 |
| LOC_Os06g43990 | expressed protein | 1.31 | 0.02 | 0.41 |
| LOC_Os10g42030 | expressed protein | 1.70 | 0.01 | 0.33 |
| LOC_Os03g03724 | expressed protein | 3.14 | 0.02 | 0.39 |
| LOC_Os01g43060 | expressed protein | -1.05 | 0.04 | 0.46 |
| LOC_Os01g71624 | expressed protein | -1.04 | 0.02 | 0.40 |
| LOC_Os01g24420 | expressed protein | 1.20 | 0.03 | 0.45 |
| LOC_Os01g42200 | expressed protein | 2.51 | 0.00 | 0.26 |
| LOC_Os05g47860 | expressed protein | -1.10 | 0.03 | 0.43 |
| LOC_Os06g50230 | expressed protein | 2.38 | 0.00 | 0.29 |
| LOC_Os05g07280 | expressed protein | 1.21 | 0.01 | 0.35 |
| LOC_Os01g48530 | expressed protein | 1.19 | 0.02 | 0.41 |
| LOC_Os01g03040 | expressed protein | -1.79 | 0.01 | 0.37 |
| LOC_Os01g07382 | expressed protein | -1.42 | 0.01 | 0.36 |
| LOC_Os04g41710 | expressed protein | 1.02 | 0.02 | 0.40 |
| LOC_Os01g40650 | expressed protein | -2.80 | 0.00 | 0.21 |
| LOC_Os12g38560 | expressed protein | -1.05 | 0.01 | 0.36 |
| LOC_Os12g05690 | expressed protein | -1.52 | 0.01 | 0.35 |
| LOC_Os01g70400 | expressed protein | -1.44 | 0.02 | 0.40 |
| LOC_Os01g07130 | expressed protein | 1.27 | 0.05 | 0.48 |
| LOC_Os05g12630 | expressed protein | -2.03 | 0.01 | 0.31 |
| LOC_Os01g05260 | expressed protein | -1.60 | 0.01 | 0.37 |
| LOC_Os03g11290 | expressed protein | -1.40 | 0.03 | 0.45 |
| LOC_Os11g02720 | expressed protein | -1.39 | 0.05 | 0.48 |
| LOC_Os09g24620 | expressed protein | -2.55 | 0.00 | 0.28 |
| LOC_Os04g27096 | expressed protein | -1.69 | 0.03 | 0.43 |
| LOC_Os03g04080 | expressed protein | -2.33 | 0.00 | 0.10 |
| LOC_Os01g01670 | expressed protein | -3.21 | 0.00 | 0.29 |
| LOC_Os01g47670 | expressed protein | -1.69 | 0.02 | 0.39 |
| LOC_Os03g02470 | expressed protein | -4.29 | 0.00 | 0.30 |
| LOC_Os04g11980 | expressed protein | -3.01 | 0.01 | 0.31 |
| LOC_Os06g04930 | expressed protein | -2.21 | 0.01 | 0.38 |
| LOC_Os06g14780 | expressed protein | -2.22 | 0.02 | 0.40 |
| LOC_Os11g01330 | expressed protein | -2.63 | 0.00 | 0.24 |
| LOC_Os01g68500 | expressed protein | -1.63 | 0.00 | 0.27 |
| LOC_Os08g43620 | expressed protein | -2.11 | 0.00 | 0.29 |
| LOC_Os01g26070 | expressed protein | -2.49 | 0.02 | 0.39 |
| LOC_Os06g11230 | expressed protein | -2.78 | 0.00 | 0.21 |
| LOC_Os11g23930 | expressed protein | -2.85 | 0.00 | 0.21 |
| LOC_Os04g33610 | expressed protein | -4.06 | 0.00 | 0.22 |
| LOC_Os11g02290 | expressed protein | -4.64 | 0.02 | 0.41 |
| LOC_Os05g48900 | fasciclin domain containing protein, expressed | -6.78 | 0.00 | 0.16 |
| LOC_Os07g23430 | fatty acid desaturase, putative, expressed | 1.21 | 0.01 | 0.35 |
| LOC_Os12g01370 | fatty acid desaturase, putative, expressed | 1.20 | 0.02 | 0.42 |
| LOC_Os04g48880 | fatty acid hydroxylase, putative, expressed | -1.48 | 0.00 | 0.21 |
| LOC_Os06g01850 | ferredoxin--NADP reductase, chloroplast precursor, putative, expressed | -1.12 | 0.01 | 0.38 |
| LOC_Os11g01530 | ferritin-1, chloroplast precursor, putative, expressed | -4.07 | 0.00 | 0.06 |
| LOC_Os08g16130 | fiber protein Fb34, putative, expressed | 1.11 | 0.01 | 0.36 |
| LOC_Os04g14710 | flavin-containing monooxygenase family protein, putative, expressed | -1.51 | 0.01 | 0.36 |
| LOC_Os05g03640 | flavonol synthase/flavanone 3-hydroxylase, putative, expressed | 1.57 | 0.00 | 0.31 |
| LOC_Os01g57460 | frataxin, putative, expressed | -1.61 | 0.01 | 0.35 |
| LOC_Os10g02584 | GAGA-binding protein, putative, expressed | 2.68 | 0.00 | 0.26 |
| LOC_Os04g38450 | gamma-glutamyltranspeptidase 1 precursor, putative, expressed | 1.13 | 0.02 | 0.41 |
| LOC_Os07g39690 | GCN5-related N-acetyltransferase, putative, expressed | -1.13 | 0.04 | 0.47 |
| LOC_Os06g04620 | GDP-mannose 4,6 dehydratase 2, putative, expressed | 4.36 | 0.00 | 0.16 |
| LOC_Os11g31940 | GDSL-like lipase/acylhydrolase, putative, expressed | 1.08 | 0.02 | 0.39 |
| LOC_Os01g58790 | GHMP kinases ATP-binding protein, putative, expressed | 1.05 | 0.03 | 0.43 |
| LOC_Os07g44890 | gibberellin receptor GID1L2, putative, expressed | -2.59 | 0.02 | 0.39 |
| LOC_Os07g35350 | glucan endo-1,3-beta-glucosidase precursor, putative, expressed | -1.49 | 0.01 | 0.33 |
| LOC_Os06g08880 | glutamate receptor 2.7 precursor, putative, expressed | 1.09 | 0.01 | 0.38 |
| LOC_Os03g50490 | glutamine synthetase, catalytic domain containing protein, expressed | -1.15 | 0.00 | 0.21 |
| LOC_Os11g14040 | glutathione S-transferase, N-terminal domain containing protein, expressed | 1.05 | 0.01 | 0.38 |
| LOC_Os01g55830 | glutathione S-transferase, putative, expressed | -1.03 | 0.03 | 0.44 |
| LOC_Os09g20220 | glutathione S-transferase, putative, expressed | -1.62 | 0.01 | 0.35 |
| LOC_Os03g03720 | glyceraldehyde-3-phosphate dehydrogenase, putative, expressed | -1.44 | 0.02 | 0.42 |
| LOC_Os05g41590 | glycerol-3-phosphate dehydrogenase, putative, expressed | -1.42 | 0.03 | 0.44 |
| LOC_Os03g40670 | glycerophosphoryl diester phosphodiesterase family protein, putative, expressed | 1.20 | 0.00 | 0.30 |
| LOC_Os10g31720 | glycine-rich cell wall structural protein 2 precursor, putative, expressed | -2.97 | 0.02 | 0.39 |
| LOC_Os01g04300 | glycosyl hydrolase family 10 protein, putative, expressed | 1.27 | 0.05 | 0.48 |
| LOC_Os11g47520 | glycosyl hydrolase, putative, expressed | -2.12 | 0.02 | 0.40 |
| LOC_Os05g31140 | glycosyl hydrolases family 17, putative, expressed | 1.92 | 0.03 | 0.44 |
| LOC_Os01g71350 | glycosyl hydrolases family 17, putative, expressed | 1.74 | 0.02 | 0.40 |
| LOC_Os04g33640 | glycosyl hydrolases family 17, putative, expressed | -2.15 | 0.02 | 0.41 |
| LOC_Os01g71670 | glycosyl hydrolases family 17, putative, expressed | -4.23 | 0.00 | 0.31 |
| LOC_Os04g33740 | glycosyl hydrolases, putative, expressed | 1.55 | 0.00 | 0.21 |
| LOC_Os06g13760 | glycosyl transferase 8 domain containing protein, putative, expressed | 3.20 | 0.00 | 0.29 |
| LOC_Os01g04920 | glycosyl transferase, group 1 domain containing protein, expressed | 1.28 | 0.00 | 0.29 |
| LOC_Os01g02900 | glycosyltransferase, putative, expressed | 1.03 | 0.02 | 0.39 |
| LOC_Os03g16940 | glyoxalase family protein, putative, expressed | -1.45 | 0.02 | 0.39 |
| LOC_Os06g19990 | GPI-anchored protein, putative, expressed | 1.26 | 0.04 | 0.47 |
| LOC_Os04g58090 | harpin-induced protein 1 domain containing protein, expressed | 6.22 | 0.00 | 0.21 |
| LOC_Os12g03370 | harpin-induced protein 1 domain containing protein, expressed | -4.36 | 0.03 | 0.44 |
| LOC_Os10g33140 | hcrVf2 protein, putative, expressed | 2.93 | 0.00 | 0.18 |
| LOC_Os03g22490 | heavy metal-associated domain containing protein, expressed | 1.19 | 0.03 | 0.42 |
| LOC_Os06g35060 | heavy metal-associated domain containing protein, expressed | 1.83 | 0.00 | 0.30 |
| LOC_Os01g48710 | heavy metal-associated domain containing protein, expressed | 1.62 | 0.04 | 0.48 |
| LOC_Os03g51580 | helix-loop-helix DNA-binding domain containing protein, expressed | 1.01 | 0.00 | 0.31 |
| LOC_Os03g53020 | helix-loop-helix DNA-binding domain containing protein, expressed | -1.13 | 0.03 | 0.44 |
| LOC_Os01g09990 | helix-loop-helix DNA-binding domain containing protein, expressed | 1.21 | 0.03 | 0.44 |
| LOC_Os05g11070 | helix-loop-helix DNA-binding domain containing protein, expressed | 1.27 | 0.02 | 0.41 |
| LOC_Os01g50940 | helix-loop-helix DNA-binding domain containing protein, expressed | -2.82 | 0.01 | 0.31 |
| LOC_Os01g64250 | hemerythrin family protein, expressed | -1.22 | 0.03 | 0.45 |
| LOC_Os03g08960 | homeobox associated leucine zipper, putative, expressed | 1.36 | 0.03 | 0.44 |
| LOC_Os10g23090 | homeobox associated leucine zipper, putative, expressed | 1.24 | 0.01 | 0.31 |
| LOC_Os01g56969 | homeobox-leucine zipper protein ATHB-54, putative, expressed | 1.34 | 0.03 | 0.46 |
| LOC_Os02g32590 | HSF-type DNA-binding domain containing protein, expressed | -2.13 | 0.03 | 0.43 |
| LOC_Os01g54550 | HSF-type DNA-binding domain containing protein, expressed | 1.63 | 0.00 | 0.30 |
| LOC_Os03g15960 | hsp20/alpha crystallin family protein, putative, expressed | 2.69 | 0.00 | 0.02 |
| LOC_Os10g07210 | hsp20/alpha crystallin family protein, putative, expressed | 1.31 | 0.04 | 0.48 |
| LOC_Os09g22000 | hydrolase, HAD superfamily, Cof family, putative, expressed | 4.24 | 0.01 | 0.38 |
| LOC_Os02g39160 | hydroxymethylbutenyl 4-diphosphate synthase, putative, expressed | 1.29 | 0.03 | 0.44 |
| LOC_Os01g58580 | ICE-like protease p20 domain containing protein, putative, expressed | 2.10 | 0.01 | 0.37 |
| LOC_Os11g07030 | integrator complex subunit 3, putative, expressed | 2.72 | 0.00 | 0.22 |
| LOC_Os06g02520 | interacting protein of DMI3, putative, expressed | 1.48 | 0.02 | 0.40 |
| LOC_Os12g18560 | invertase/pectin methylesterase inhibitor family protein, putative, expressed | 3.31 | 0.03 | 0.43 |
| LOC_Os07g34520 | isocitrate lyase, putative, expressed | -1.95 | 0.03 | 0.45 |
| LOC_Os01g01660 | isoflavone reductase, putative, expressed | -4.86 | 0.02 | 0.42 |
| LOC_Os04g24328 | jasmonate-induced protein, putative, expressed | 1.49 | 0.05 | 0.48 |
| LOC_Os01g61160 | laccase precursor protein, putative, expressed | 1.18 | 0.01 | 0.37 |
| LOC_Os07g02810 | L-ascorbate oxidase homolog precursor, putative, expressed | -1.26 | 0.04 | 0.48 |
| LOC_Os03g06360 | late embryogenesis abundant protein D-34, putative, expressed | -2.14 | 0.01 | 0.35 |
| LOC_Os04g51580 | leucine rich repeat containing protein, expressed | 2.50 | 0.03 | 0.43 |
| LOC_Os03g09070 | leucine rich repeat domain containing protein, putative, expressed | -1.36 | 0.05 | 0.48 |
| LOC_Os12g10740 | leucine-rich repeat family protein, putative, expressed | 1.23 | 0.01 | 0.38 |
| LOC_Os04g45170 | leucine-rich repeat family protein, putative, expressed | 1.23 | 0.01 | 0.32 |
| LOC_Os03g52860 | lipoxygenase, putative, expressed | 1.23 | 0.02 | 0.39 |
| LOC_Os05g39230 | low photochemical bleaching 1 protein, putative, expressed | 1.28 | 0.00 | 0.26 |
| LOC_Os06g46390 | LSM domain containing protein, expressed | -1.16 | 0.04 | 0.46 |
| LOC_Os04g33920 | LTPL102 - Protease inhibitor/seed storage/LTP family protein precursor, expressed | -1.61 | 0.03 | 0.43 |
| LOC_Os05g06780 | LTPL104 - Protease inhibitor/seed storage/LTP family protein precursor, expressed | 1.65 | 0.00 | 0.26 |
| LOC_Os03g50960 | LTPL118 - Protease inhibitor/seed storage/LTP family protein precursor, expressed | -1.29 | 0.03 | 0.43 |
| LOC_Os10g40470 | LTPL142 - Protease inhibitor/seed storage/LTP family protein precursor, putative, expressed | 1.55 | 0.00 | 0.21 |
| LOC_Os10g40510 | LTPL144 - Protease inhibitor/seed storage/LTP family protein precursor, expressed | -1.98 | 0.01 | 0.39 |
| LOC_Os03g02050 | LTPL151 - Protease inhibitor/seed storage/LTP family protein precursor, expressed | -2.80 | 0.02 | 0.41 |
| LOC_Os08g03690 | LTPL24 - Protease inhibitor/seed storage/LTP family protein precursor, expressed | -2.76 | 0.03 | 0.43 |
| LOC_Os03g26820 | LTPL52 - Protease inhibitor/seed storage/LTP family protein precursor, expressed | -1.18 | 0.04 | 0.48 |
| LOC_Os07g07930 | LTPL78 - Protease inhibitor/seed storage/LTP family protein precursor, expressed | -1.54 | 0.00 | 0.31 |
| LOC_Os06g04130 | lung seven transmembrane domain containing protein, putative, expressed | -3.20 | 0.02 | 0.40 |
| LOC_Os07g44650 | LYR motif containing protein, putative, expressed | -3.46 | 0.05 | 0.48 |
| LOC_Os01g17170 | magnesium-protoporphyrin IX monomethyl ester cyclase,chloroplast precursor, putative, expressed | -1.14 | 0.05 | 0.48 |
| LOC_Os11g16530 | mal, putative, expressed | 1.06 | 0.01 | 0.33 |
| LOC_Os07g31884 | MATE efflux family protein, putative, expressed | -1.03 | 0.03 | 0.44 |
| LOC_Os04g30490 | MATE efflux family protein, putative, expressed | -1.21 | 0.00 | 0.22 |
| LOC_Os04g48290 | MATE efflux family protein, putative, expressed | -1.13 | 0.04 | 0.48 |
| LOC_Os07g07270 | MBTB13 - Bric-a-Brac, Tramtrack, Broad Complex BTB domain with Meprin and TRAF Homology MATH domain, expressed | -1.05 | 0.02 | 0.41 |
| LOC_Os04g33830 | membrane protein, putative, expressed | -2.03 | 0.01 | 0.37 |
| LOC_Os03g15910 | membrane protein, putative, expressed | -1.37 | 0.01 | 0.35 |
| LOC_Os01g74110 | metal cation transporter, putative, expressed | 2.20 | 0.02 | 0.41 |
| LOC_Os07g15370 | metal transporter Nramp6, putative, expressed | 1.16 | 0.01 | 0.34 |
| LOC_Os03g63590 | metallo-beta-lactamase, putative, expressed | -1.34 | 0.03 | 0.45 |
| LOC_Os06g13180 | metalloendoproteinase 1 precursor, putative, expressed | 2.28 | 0.02 | 0.42 |
| LOC_Os01g05650 | metallothionein, putative, expressed | -1.52 | 0.00 | 0.26 |
| LOC_Os12g38300 | metallothionein, putative, expressed | 1.51 | 0.01 | 0.36 |
| LOC_Os10g10180 | methyltransferase domain containing protein, putative, expressed | -1.54 | 0.02 | 0.39 |
| LOC_Os01g51870 | methyltransferase, putative, expressed | -1.09 | 0.01 | 0.34 |
| LOC_Os01g67360 | methyltransferase, putative, expressed | 1.29 | 0.01 | 0.35 |
| LOC_Os08g20420 | MGD2, putative, expressed | 1.70 | 0.02 | 0.41 |
| LOC_Os11g01270 | mitochondrial substrate carrier family protein, putative, expressed | -2.02 | 0.04 | 0.48 |
| LOC_Os03g57420 | ML domain protein, putative, expressed | -1.06 | 0.01 | 0.34 |
| LOC_Os07g30960 | monooxygenase, putative, expressed | -1.42 | 0.00 | 0.29 |
| LOC_Os01g10350 | MPPN domain containing protein, expressed | -1.26 | 0.03 | 0.44 |
| LOC_Os12g01510 | MRH1, putative, expressed | 1.80 | 0.00 | 0.29 |
| LOC_Os02g34630 | MYB family transcription factor, putative, expressed | 1.78 | 0.00 | 0.30 |
| LOC_Os11g01480 | MYB family transcription factor, putative, expressed | -2.68 | 0.01 | 0.35 |
| LOC_Os03g20900 | Myb transcription factor, putative, expressed | -1.35 | 0.04 | 0.48 |
| LOC_Os06g24070 | myb-like DNA-binding domain containing protein, expressed | -1.12 | 0.01 | 0.34 |
| LOC_Os02g09480 | myb-like DNA-binding domain containing protein, putative, expressed | 3.82 | 0.00 | 0.26 |
| LOC_Os06g05130 | myristoyl-acyl carrier protein thioesterase, chloroplast precursor, putative, expressed | -1.02 | 0.01 | 0.36 |
| LOC_Os01g54030 | NADP-dependent malic enzyme, putative, expressed | -1.25 | 0.03 | 0.44 |
| LOC_Os12g12590 | NADP-dependent oxidoreductase, putative, expressed | 1.95 | 0.01 | 0.36 |
| LOC_Os02g50940 | NHL repeat-containing protein, putative, expressed | 4.16 | 0.00 | 0.30 |
| LOC_Os09g37710 | NIN, putative, expressed | 1.16 | 0.01 | 0.37 |
| LOC_Os02g53130 | nitrate reductase, putative, expressed | -1.15 | 0.03 | 0.44 |
| LOC_Os07g48450 | no apical meristem protein, putative, expressed | 1.54 | 0.01 | 0.36 |
| LOC_Os01g23710 | no apical meristem protein, putative, expressed | 1.37 | 0.01 | 0.31 |
| LOC_Os04g55850 | nuclease PA3, putative, expressed | 1.18 | 0.01 | 0.36 |
| LOC_Os07g37110 | nucleoside transporter, putative, expressed | 1.41 | 0.03 | 0.44 |
| LOC_Os12g38750 | nucleotide pyrophosphatase/phosphodiesterase, putative, expressed | 1.34 | 0.03 | 0.44 |
| LOC_Os11g01340 | omega-3 fatty acid desaturase, chloroplast precursor, putative, expressed | -2.49 | 0.00 | 0.27 |
| LOC_Os01g70520 | Os1bglu5 - beta-glucosidase homologue, similar to G. max isohydroxyurate hydrolase, expressed | -1.12 | 0.02 | 0.42 |
| LOC_Os02g51850 | OsAPRL3 adenosine 5'-phosphosulfate reductase-like OsAPRL3, expressed | -1.57 | 0.00 | 0.18 |
| LOC_Os11g11210 | OsClp12 - Putative Clp protease homologue, expressed | -1.80 | 0.02 | 0.39 |
| LOC_Os03g21380 | OsCML27 - Calmodulin-related calcium sensor protein, expressed | 1.52 | 0.02 | 0.39 |
| LOC_Os02g21260 | OsFBL8 - F-box domain and LRR containing protein, expressed | -1.77 | 0.05 | 0.48 |
| LOC_Os07g37400 | OsFBX257 - F-box domain containing protein, expressed | -1.95 | 0.01 | 0.38 |
| LOC_Os04g42930 | OsGrx_C2.2 - glutaredoxin subgroup I, expressed | -1.06 | 0.01 | 0.36 |
| LOC_Os03g17790 | OsRCI2-5 - Putative low temperature and salt responsive protein, expressed | -1.78 | 0.01 | 0.34 |
| LOC_Os11g24340 | OsSCP53 - Putative Serine Carboxypeptidase homologue, expressed | 1.41 | 0.01 | 0.38 |
| LOC_Os03g55350 | OsSub31 - Putative Subtilisin homologue, expressed | 4.08 | 0.00 | 0.21 |
| LOC_Os11g01490 | outer membrane protein, OMP85 family, putative, expressed | -1.87 | 0.00 | 0.29 |
| LOC_Os04g37490 | oxidoreductase, aldo/keto reductase family protein, putative, expressed | -1.34 | 0.03 | 0.43 |
| LOC_Os10g35370 | oxidoreductase, short chain dehydrogenase/reductase family domain containing family, expressed | -1.31 | 0.02 | 0.40 |
| LOC_Os06g10510 | oxidoreductase/ transition metal ion binding protein, putative, expressed | 2.44 | 0.04 | 0.48 |
| LOC_Os07g36080 | oxygen evolving enhancer protein 3 domain containing protein, expressed | -1.01 | 0.03 | 0.45 |
| LOC_Os01g31690 | oxygen-evolving enhancer protein 1, chloroplast precursor, putative, expressed | -1.33 | 0.01 | 0.36 |
| LOC_Os10g42500 | PAP fibrillin family domain containing protein, expressed | -1.71 | 0.03 | 0.45 |
| LOC_Os12g36850 | pathogenesis-related Bet v I family protein, putative, expressed | 2.08 | 0.01 | 0.37 |
| LOC_Os07g44070 | pectinacetylesterase domain containing protein, expressed | -1.13 | 0.00 | 0.28 |
| LOC_Os06g07878 | peptidase, T1 family, putative, expressed | 3.38 | 0.03 | 0.43 |
| LOC_Os05g33510 | peptide methionine sulfoxide reductase msrB, putative, expressed | -1.65 | 0.05 | 0.48 |
| LOC_Os10g40600 | peptide transporter PTR2, putative, expressed | 1.42 | 0.00 | 0.22 |
| LOC_Os01g37590 | peptide transporter PTR2, putative, expressed | -2.02 | 0.01 | 0.34 |
| LOC_Os01g38359 | peptidyl-prolyl cis-trans isomerase, FKBP-type, putative, expressed | 1.46 | 0.02 | 0.41 |
| LOC_Os06g49470 | peptidyl-prolyl cis-trans isomerase, putative, expressed | -1.28 | 0.01 | 0.33 |
| LOC_Os04g28420 | peptidyl-prolyl isomerase, putative, expressed | -1.03 | 0.01 | 0.34 |
| LOC_Os04g39100 | peroxidase precursor, putative, expressed | 3.52 | 0.00 | 0.29 |
| LOC_Os01g73170 | peroxidase precursor, putative, expressed | 1.67 | 0.00 | 0.29 |
| LOC_Os04g59160 | peroxidase precursor, putative, expressed | 1.23 | 0.04 | 0.48 |
| LOC_Os07g48030 | peroxidase precursor, putative, expressed | 1.80 | 0.05 | 0.48 |
| LOC_Os06g09610 | peroxiredoxin, putative, expressed | -1.53 | 0.05 | 0.48 |
| LOC_Os07g44430 | peroxiredoxin, putative, expressed | -3.30 | 0.01 | 0.35 |
| LOC_Os01g07730 | phosphate/phosphoenolpyruvate translocator-related protein, putative, expressed | 1.28 | 0.04 | 0.46 |
| LOC_Os02g52000 | phosphate-induced protein 1 conserved region domain containing protein, expressed | 3.72 | 0.00 | 0.22 |
| LOC_Os12g01480 | phosphatidate cytidylyltransferase, putative, expressed | 1.95 | 0.00 | 0.30 |
| LOC_Os11g01450 | phosphatidate cytidylyltransferase, putative, expressed | -2.88 | 0.02 | 0.40 |
| LOC_Os06g46900 | phosphosulfolactate synthase-related protein, putative, expressed | -1.17 | 0.00 | 0.21 |
| LOC_Os08g44680 | photosystem I reaction center subunit II, chloroplast precursor, putative, expressed | -1.45 | 0.00 | 0.06 |
| LOC_Os12g08770 | photosystem I reaction center subunit N, chloroplast precursor, putative, expressed | -1.04 | 0.00 | 0.21 |
| LOC_Os12g23200 | photosystem I reaction center subunit XI, chloroplast precursor, putative, expressed | -1.09 | 0.00 | 0.23 |
| LOC_Os07g05480 | photosystem I reaction center subunit, chloroplast precursor, putative, expressed | -2.17 | 0.00 | 0.28 |
| LOC_Os08g10020 | photosystem II 10 kDa polypeptide, chloroplast precursor, putative, expressed | -1.94 | 0.00 | 0.28 |
| LOC_Os01g56680 | photosystem II reaction center W protein, chloroplast precursor, putative, expressed | -3.67 | 0.03 | 0.45 |
| LOC_Os03g52390 | PIII1 - Proteinase inhibitor II family protein precursor, expressed | -4.39 | 0.01 | 0.38 |
| LOC_Os03g52360 | PIII3 - Proteinase inhibitor II family protein precursor, putative, expressed | -2.01 | 0.04 | 0.46 |
| LOC_Os11g25040 | plant-specific domain TIGR01615 family protein, expressed | 1.95 | 0.04 | 0.46 |
| LOC_Os11g34940 | plastid-specific 50S ribosomal protein 6, chloroplast precursor, putative, expressed | -3.45 | 0.00 | 0.24 |
| LOC_Os12g01560 | PMR5, putative, expressed | 1.53 | 0.04 | 0.46 |
| LOC_Os11g01370 | PMR5, putative, expressed | -1.08 | 0.01 | 0.36 |
| LOC_Os07g47750 | POEI49 - Pollen Ole e I allergen and extensin family protein precursor, expressed | 2.01 | 0.00 | 0.30 |
| LOC_Os02g32860 | poly synthetase 3, putative, expressed | -1.10 | 0.04 | 0.48 |
| LOC_Os05g50550 | polyprenyl synthetase, putative, expressed | -1.24 | 0.03 | 0.44 |
| LOC_Os07g12140 | PPR repeat domain containing protein, putative, expressed | -1.49 | 0.02 | 0.40 |
| LOC_Os12g18110 | PQ loop repeat domain containing protein, expressed | 1.08 | 0.03 | 0.44 |
| LOC_Os01g28790 | PRAS-rich protein, putative, expressed | -5.87 | 0.00 | 0.28 |
| LOC_Os05g16930 | protein kinase domain containing protein, expressed | 1.52 | 0.01 | 0.37 |
| LOC_Os03g28300 | protein kinase domain containing protein, expressed | 1.35 | 0.00 | 0.27 |
| LOC_Os02g35910 | protein phosphotase protein, putative, expressed | -1.04 | 0.01 | 0.37 |
| LOC_Os04g58200 | protochlorophyllide reductase A, chloroplast precursor, putative, expressed | -2.06 | 0.02 | 0.42 |
| LOC_Os10g41999 | RALFL27 - Rapid ALkalinization Factor RALF family protein precursor, expressed | 1.72 | 0.00 | 0.28 |
| LOC_Os01g15320 | RALFL9 - Rapid ALkalinization Factor RALF family protein precursor, expressed | -2.76 | 0.03 | 0.43 |
| LOC_Os06g35814 | ras-related protein, putative, expressed | 5.73 | 0.01 | 0.34 |
| LOC_Os08g41340 | ras-related protein, putative, expressed | -1.22 | 0.01 | 0.34 |
| LOC_Os03g62600 | ras-related protein, putative, expressed | 1.36 | 0.04 | 0.46 |
| LOC_Os11g05360 | RCLEA9 - Root cap and Late embryogenesis related family protein precursor, putative, expressed | -1.91 | 0.02 | 0.40 |
| LOC_Os10g35040 | receptor kinase like protein, putative, expressed | 1.41 | 0.03 | 0.43 |
| LOC_Os12g08180 | receptor-like protein kinase 2 precursor, putative, expressed | 1.60 | 0.01 | 0.37 |
| LOC_Os03g03290 | receptor-like protein kinase At3g46290 precursor, putative, expressed | -2.92 | 0.00 | 0.28 |
| LOC_Os10g25040 | red chlorophyll catabolite reductase, putative, expressed | 1.46 | 0.00 | 0.28 |
| LOC_Os03g02040 | remorin, putative, expressed | 1.09 | 0.01 | 0.34 |
| LOC_Os04g56730 | repressor of RNA polymerase III transcription MAF1, putative, expressed | 1.40 | 0.01 | 0.38 |
| LOC_Os09g36220 | response regulator receiver domain containing protein, expressed | -1.31 | 0.00 | 0.30 |
| LOC_Os07g23640 | retrotransposon protein, putative, Ty3-gypsy subclass, expressed | -1.05 | 0.00 | 0.21 |
| LOC_Os07g04950 | retrotransposon protein, putative, unclassified, expressed | 1.37 | 0.02 | 0.42 |
| LOC_Os12g24020 | rhodanese-like domain containing protein, putative, expressed | 1.50 | 0.04 | 0.48 |
| LOC_Os09g36040 | rhodanese-like domain containing protein, putative, expressed | -1.08 | 0.01 | 0.36 |
| LOC_Os11g01420 | ribosomal protein L10, putative, expressed | -4.51 | 0.00 | 0.21 |
| LOC_Os01g59730 | ribosomal protein L7Ae, putative, expressed | -1.14 | 0.02 | 0.39 |
| LOC_Os12g19381 | ribulose bisphosphate carboxylase small chain, chloroplast precursor, putative, expressed | -1.41 | 0.01 | 0.35 |
| LOC_Os04g48310 | RING-H2 finger protein, putative, expressed | -1.09 | 0.02 | 0.42 |
| LOC_Os01g60730 | RING-H2 finger protein, putative, expressed | 1.15 | 0.01 | 0.36 |
| LOC_Os02g46100 | RING-H2 finger protein, putative, expressed | -2.13 | 0.02 | 0.40 |
| LOC_Os12g01190 | RNA recognition motif containing protein, putative, expressed | 1.20 | 0.00 | 0.21 |
| LOC_Os12g01010 | RNA recognition motif containing protein, putative, expressed | 1.17 | 0.00 | 0.30 |
| LOC_Os08g23120 | RNA recognition motif containing protein, putative, expressed | 1.62 | 0.01 | 0.38 |
| LOC_Os03g17060 | RNA recognition motif containing protein, putative, expressed | -2.01 | 0.04 | 0.46 |
| LOC_Os12g07680 | RNase P subunit p30, putative, expressed | 1.59 | 0.04 | 0.46 |
| LOC_Os02g39795 | S-adenosyl-l-methionine decarboxylase leader peptide, putative, expressed | 1.31 | 0.01 | 0.34 |
| LOC_Os06g22440 | SAM dependent carboxyl methyltransferase, putative, expressed | 1.81 | 0.02 | 0.42 |
| LOC_Os06g20790 | SAM dependent carboxyl methyltransferase, putative, expressed | 1.49 | 0.02 | 0.39 |
| LOC_Os10g11500 | SCP-like extracellular protein, expressed | 8.49 | 0.00 | 0.17 |
| LOC_Os12g01200 | senescence-induced receptor-like serine/threonine-protein kinase precursor, putative, expressed | 1.12 | 0.03 | 0.44 |
| LOC_Os11g01200 | senescence-induced receptor-like serine/threonine-protein kinase precursor, putative, expressed | -2.19 | 0.01 | 0.33 |
| LOC_Os07g46846 | sex determination protein tasselseed-2, putative, expressed | 1.90 | 0.02 | 0.41 |
| LOC_Os10g35840 | shikimate/quinate 5-dehydrogenase, putative, expressed | -1.39 | 0.03 | 0.44 |
| LOC_Os07g07540 | SHOOT1 protein, putative, expressed | -1.05 | 0.00 | 0.31 |
| LOC_Os05g28210 | small hydrophilic plant seed protein, putative, expressed | -2.72 | 0.01 | 0.36 |
| LOC_Os01g64670 | soluble inorganic pyrophosphatase, putative, expressed | -1.92 | 0.03 | 0.43 |
| LOC_Os05g36260 | soluble inorganic pyrophosphatase, putative, expressed | -1.05 | 0.01 | 0.34 |
| LOC_Os02g33020 | SOUL heme-binding protein, putative, expressed | -1.12 | 0.05 | 0.48 |
| LOC_Os02g04950 | splicing factor 3B subunit 1, putative, expressed | -1.80 | 0.02 | 0.41 |
| LOC_Os12g02250 | STE_PAK_Ste20_Slob_Wnk.3 - STE kinases include homologs to sterile 7, sterile 11 and sterile 20 from yeast, expressed | 1.10 | 0.02 | 0.42 |
| LOC_Os03g21040 | stress responsive protein, putative, expressed | -1.46 | 0.02 | 0.40 |
| LOC_Os11g34920 | stripe rust resistance protein Yr10, putative, expressed | 1.20 | 0.00 | 0.30 |
| LOC_Os11g37860 | stripe rust resistance protein Yr10, putative, expressed | 1.43 | 0.02 | 0.41 |
| LOC_Os03g09970 | sulfate transporter, putative, expressed | 1.54 | 0.02 | 0.41 |
| LOC_Os05g25850 | superoxide dismutase, mitochondrial precursor, putative, expressed | -1.08 | 0.02 | 0.41 |
| LOC_Os08g40420 | ternary complex factor MIP1, putative, expressed | 1.05 | 0.03 | 0.44 |
| LOC_Os11g28530 | terpene synthase, putative, expressed | 1.22 | 0.03 | 0.42 |
| LOC_Os03g24690 | terpene synthase, putative, expressed | 3.13 | 0.00 | 0.29 |
| LOC_Os03g14030 | thaumatin, putative, expressed | 1.16 | 0.01 | 0.37 |
| LOC_Os03g45960 | thaumatin, putative, expressed | -2.78 | 0.00 | 0.29 |
| LOC_Os12g26960 | THION34 - Plant thionin family protein precursor, expressed | 4.65 | 0.00 | 0.30 |
| LOC_Os06g32160 | THION7 - Plant thionin family protein precursor, expressed | 1.80 | 0.02 | 0.42 |
| LOC_Os04g57930 | thioredoxin, putative, expressed | -2.20 | 0.02 | 0.41 |
| LOC_Os03g17300 | TKL_IRAK_CrRLK1L-1.7 - The CrRLK1L-1 subfamily has homology to the CrRLK1L homolog, expressed | -2.72 | 0.01 | 0.34 |
| LOC_Os07g35370 | TKL_IRAK_DUF26-lc.15 - DUF26 kinases have homology to DUF26 containing loci, expressed | 1.42 | 0.02 | 0.41 |
| LOC_Os05g23950 | TRAF-type zinc finger family protein, expressed | -1.17 | 0.01 | 0.36 |
| LOC_Os02g35690 | transcription factor like protein, putative, expressed | 3.25 | 0.00 | 0.26 |
| LOC_Os11g05480 | transcription factor, putative, expressed | 1.22 | 0.00 | 0.21 |
| LOC_Os02g57480 | transferase family protein, putative, expressed | 3.24 | 0.00 | 0.21 |
| LOC_Os10g35950 | transferase family protein, putative, expressed | 1.83 | 0.00 | 0.26 |
| LOC_Os07g09190 | transketolase, putative, expressed | 2.50 | 0.01 | 0.38 |
| LOC_Os10g39440 | transporter family protein, putative, expressed | 1.25 | 0.03 | 0.45 |
| LOC_Os03g39710 | transporter family protein, putative, expressed | 1.10 | 0.02 | 0.42 |
| LOC_Os04g37980 | transporter family protein, putative, expressed | 1.06 | 0.03 | 0.45 |
| LOC_Os04g37990 | transporter family protein, putative, expressed | 1.19 | 0.01 | 0.36 |
| LOC_Os04g46880 | transporter, major facilitator family, putative, expressed | 1.47 | 0.04 | 0.47 |
| LOC_Os01g17240 | transporter, major facilitator family, putative, expressed | -1.21 | 0.00 | 0.24 |
| LOC_Os02g42220 | transposon protein, putative, unclassified, expressed | 1.18 | 0.04 | 0.48 |
| LOC_Os06g08530 | ubiquitin carboxyl-terminal hydrolase domain containing protein, expressed | 1.04 | 0.05 | 0.48 |
| LOC_Os03g15370 | ubiquitin fusion protein, putative, expressed | -1.89 | 0.00 | 0.30 |
| LOC_Os04g58800 | ubiquitin-conjugating enzyme, putative, expressed | -1.16 | 0.03 | 0.45 |
| LOC_Os10g40120 | U-box domain containing protein, expressed | 1.08 | 0.00 | 0.30 |
| LOC_Os05g08750 | UDP-glucoronosyl and UDP-glucosyl transferase domain containing protein, expressed | -2.13 | 0.02 | 0.41 |
| LOC_Os04g12970 | UDP-glucoronosyl/UDP-glucosyl transferase, putative, expressed | -2.52 | 0.01 | 0.33 |
| LOC_Os11g38650 | UDP-glucoronosyl/UDP-glucosyl transferase, putative, expressed | -3.44 | 0.01 | 0.38 |
| LOC_Os09g20390 | uncharacterized glycosyl hydrolase Rv2006/MT2062, putative, expressed | -1.14 | 0.03 | 0.44 |
| LOC_Os07g43160 | uncharacterized glycosyl hydrolase Rv2006/MT2062, putative, expressed | 1.25 | 0.01 | 0.36 |
| LOC_Os02g30460 | uncharacterized protein MJ0304, putative, expressed | -3.18 | 0.04 | 0.48 |
| LOC_Os07g37310 | uncharacterized secreted protein, putative, expressed | -1.56 | 0.01 | 0.37 |
| LOC_Os02g03710 | UP-9A, putative, expressed | -3.04 | 0.00 | 0.21 |
| LOC_Os01g09080 | WRKY107, expressed | 1.20 | 0.01 | 0.38 |
| LOC_Os05g25770 | WRKY45, expressed | 1.61 | 0.02 | 0.40 |
| LOC_Os09g25070 | WRKY62, expressed | 1.47 | 0.04 | 0.47 |
| LOC_Os04g51560 | WRKY68, expressed | -2.09 | 0.01 | 0.33 |
| LOC_Os01g53650 | zinc finger CCCH type family protein, putative, expressed | -1.08 | 0.02 | 0.41 |
| LOC_Os02g58440 | zinc finger C-x8-C-x5-C-x3-H type family protein, expressed | 3.16 | 0.02 | 0.39 |
| LOC_Os01g49280 | zinc finger family protein, putative, expressed | 1.15 | 0.01 | 0.34 |
| LOC_Os01g59980 | zinc finger family protein, putative, expressed | -1.24 | 0.05 | 0.48 |
| LOC_Os05g39380 | zinc finger, C3HC4 type domain containing protein, expressed | 2.48 | 0.01 | 0.34 |
| LOC_Os03g15000 | Zinc finger, C3HC4 type domain containing protein, expressed | 1.05 | 0.03 | 0.45 |
| LOC_Os06g34530 | zinc finger, C3HC4 type domain containing protein, expressed | -2.18 | 0.01 | 0.37 |
| LOC_Os04g59580 | zinc finger, C3HC4 type domain containing protein, expressed | -2.29 | 0.01 | 0.31 |
| LOC_Os04g40090 | zinc finger, ZZ type family protein, expressed | 1.11 | 0.01 | 0.31 |
| LOC_Os03g60560 | ZOS3-21 - C2H2 zinc finger protein, expressed | -5.57 | 0.01 | 0.34 |
| LOC_Os09g25430 | ZOS9-07 - C2H2 zinc finger protein, expressed | -1.14 | 0.04 | 0.46 |
