# Supplementary material for: Suppression of microRNA168 enhances salt tolerance in rice (Oryza sativa L.)
Source: BMC Plant Biol. 2022 Dec 3;22:563. doi: 10.1186/s12870-022-03959-1 (PMC9719116; doi:10.1186/s12870-022-03959-1)
Supplement: Supplementary file 3 — Table S3. Stress-responsive genes differentially expressed between the roots of CK and STTM168 plants grown in salt. [file 12870_2022_3959_MOESM3_ESM.docx]

**Supplementary Table 3** Stress-responsive genes differentially expressed between the roots of CK and STTM168 plants grown in salt

| Gene name | Description | log2(fc) | P-val | Q-val |
| --- | --- | --- | --- | --- |
| LOC_Os06g35814 | ras-related protein, putative, expressed | 5.73 | 0.01 | 0.34 |
| LOC_Os02g03840 | expressed protein | 1.09 | 0.01 | 0.33 |
| LOC_Os03g17790 | OsRCI2-5 - Putative low temperature and salt responsive protein, expressed | -1.78 | 0.01 | 0.34 |
| LOC_Os03g50540 | 2Fe-2S iron-sulfur cluster binding domain containing protein, expressed | 1.62 | 0.01 | 0.31 |
| LOC_Os04g15920 | dehydrogenase, putative, expressed | 1.89 | 0.01 | 0.36 |
| LOC_Os08g41340 | ras-related protein, putative, expressed | -1.22 | 0.01 | 0.34 |
| LOC_Os10g35840 | shikimate/quinate 5-dehydrogenase, putative, expressed | -1.39 | 0.03 | 0.44 |
| LOC_Os01g72900 | abscisic stress-ripening, putative, expressed | -1.53 | 0.03 | 0.43 |
| LOC_Os06g12310 | aquaporin protein, putative, expressed | 2.09 | 0.03 | 0.43 |
| LOC_Os02g51110 | aquaporin protein, putative, expressed | 1.68 | 0.03 | 0.44 |
| LOC_Os10g35050 | aquaporin protein, putative, expressed | -1.73 | 0.02 | 0.40 |
| LOC_Os03g10110 | cupin domain containing protein, expressed | -2.67 | 0.00 | 0.28 |
| LOC_Os10g21670 | dehydration stress-induced protein, putative, expressed | 1.94 | 0.04 | 0.48 |
| LOC_Os11g26790 | dehydrin, putative, expressed | -1.96 | 0.05 | 0.48 |
| LOC_Os11g40690 | dehydrogenase, putative, expressed | 3.61 | 0.00 | 0.28 |
| LOC_Os08g01760 | dehydrogenase, putative, expressed | 1.06 | 0.04 | 0.46 |
| LOC_Os10g29470 | dehydrogenase, putative, expressed | -1.50 | 0.04 | 0.46 |
| LOC_Os06g50230 | expressed protein | 2.38 | 0.00 | 0.29 |
| LOC_Os04g38450 | gamma-glutamyltranspeptidase 1 precursor, putative, expressed | 1.13 | 0.02 | 0.41 |
| LOC_Os11g14040 | glutathione S-transferase, N-terminal domain containing protein, expressed | 1.05 | 0.01 | 0.38 |
| LOC_Os01g55830 | glutathione S-transferase, putative, expressed | -1.03 | 0.03 | 0.44 |
| LOC_Os09g20220 | glutathione S-transferase, putative, expressed | -1.62 | 0.01 | 0.35 |
| LOC_Os03g03720 | glyceraldehyde-3-phosphate dehydrogenase, putative, expressed | -1.44 | 0.02 | 0.42 |
| LOC_Os05g41590 | glycerol-3-phosphate dehydrogenase, putative, expressed | -1.42 | 0.03 | 0.44 |
| LOC_Os01g54550 | HSF-type DNA-binding domain containing protein, expressed | 1.63 | 0.00 | 0.30 |
| LOC_Os02g32590 | HSF-type DNA-binding domain containing protein, expressed | -2.13 | 0.03 | 0.43 |
| LOC_Os03g15960 | hsp20/alpha crystallin family protein, putative, expressed | 2.69 | 0.00 | 0.02 |
| LOC_Os03g06360 | late embryogenesis abundant protein D-34, putative, expressed | -2.14 | 0.01 | 0.35 |
| LOC_Os04g51580 | leucine rich repeat containing protein, expressed | 2.50 | 0.03 | 0.43 |
| LOC_Os07g07270 | MBTB13 - Bric-a-Brac, Tramtrack, Broad Complex BTB domain with Meprin and TRAF Homology MATH domain, expressed | -1.05 | 0.02 | 0.41 |
| LOC_Os01g05650 | metallothionein, putative, expressed | -1.52 | 0.00 | 0.26 |
| LOC_Os02g09480 | myb-like DNA-binding domain containing protein, putative, expressed | 3.82 | 0.00 | 0.26 |
| LOC_Os01g54030 | NADP-dependent malic enzyme, putative, expressed | -1.25 | 0.03 | 0.44 |
| LOC_Os12g12590 | NADP-dependent oxidoreductase, putative, expressed | 1.95 | 0.01 | 0.36 |
| LOC_Os10g35370 | oxidoreductase, short chain dehydrogenase/reductase family domain containing family, expressed | -1.31 | 0.02 | 0.40 |
| LOC_Os04g28420 | peptidyl-prolyl isomerase, putative, expressed | -1.03 | 0.01 | 0.34 |
| LOC_Os07g44430 | peroxiredoxin, putative, expressed | -3.30 | 0.01 | 0.35 |
| LOC_Os03g28300 | protein kinase domain containing protein, expressed | 1.35 | 0.00 | 0.27 |
| LOC_Os03g62600 | ras-related protein, putative, expressed | 1.36 | 0.04 | 0.46 |
| LOC_Os11g05360 | RCLEA9 - Root cap and Late embryogenesis related family protein precursor, putative, expressed | -1.91 | 0.02 | 0.40 |
| LOC_Os03g17060 | RNA recognition motif containing protein, putative, expressed | -2.01 | 0.04 | 0.46 |
| LOC_Os03g21040 | stress responsive protein, putative, expressed | -1.46 | 0.02 | 0.40 |
| LOC_Os05g25850 | superoxide dismutase, mitochondrial precursor, putative, expressed | -1.08 | 0.02 | 0.41 |
| LOC_Os03g60560 | ZOS3-21 - C2H2 zinc finger protein, expressed | -5.57 | 0.01 | 0.34 |
